# Characterization of an immunocompetent, young adult mouse model for studying chikungunya virus neuroinvasion and central nervous system infection

**DOI:** 10.1371/journal.ppat.1014395

**Published:** 2026-07-10

**Authors:** Alyssa M. Lantz, Freedom M. Green, Amanda J. Cowan, Kestrel A. Miller, Reina A. Saldivar, Elizabeth J. Anderson, Ramya S. Barre, Vinay Shivanna, William B. Klimstra, Luis Martinez-Sobrido, Victoria K. Baxter

**Affiliations:** 1 Texas Biomedical Research Institute, San Antonio, Texas, United States of America; 2 South Texas Medical Scientist Training Program, University of Texas at San Antonio, San Antonio, Texas, United States of America; 3 Department of Microbiology, Immunology, and Genetics, University of Texas at San Antonio, San Antonio, Texas, United States of America; 4 Division of Comparative Medicine, University of North Carolina at Chapel Hill, Chapel Hill, North Carolina, United States of America; 5 Center for Vaccine Research, Department of Immunology, University of Pittsburgh, Pittsburgh, Pennsylvania, United States of America; National Institute of Allergy and Infectious Diseases Laboratory of Viral Diseases, UNITED STATES OF AMERICA

## Abstract

The arthropod-borne chikungunya virus poses a re-emerging global health threat, causing millions of cases worldwide. Neurological complications induced by chikungunya virus are being increasingly reported in vulnerable populations, including infants and young children. However, the mechanisms by which chikungunya virus invades the central nervous system and drives prolonged neurological dysfunction remain poorly defined. Studying neurological chikungunya virus infection has been limited by the lack of an immunocompetent, neurodevelopmentally appropriate small-animal model that reliably develops central nervous system infection and neurological disease by infection routes analogous to natural transmission. Following a screen of ten Collaborative Cross mouse strains, we identified four-to-six-week-old CC041 mice as an immunocompetent, neurodevelopmentally appropriate model that consistently exhibits chikungunya virus neuroinvasion and clinical signs consistent with neurological disease following peripheral inoculation. Central nervous system infection in CC041 mice was dependent on the chikungunya viral strain used, despite comparable viral replication at the inoculation site, and occurred at physiologically relevant inoculation doses without enhancement at higher doses. Comparative analyses with neuroinvasion-resistant C57BL/6J mice demonstrated that susceptibility to neuroinvasion in CC041 mice was associated with enhanced dissemination beyond the site of inoculation, prolonged serum viral load, and lower peripheral type I interferon levels early in infection. In vivo imaging using a reporter chikungunya virus confirmed rapid viral spread and early brain localization in CC041 mice, in contrast to the restricted peripheral infection observed in C57BL/6J mice. Examination of viral antigen expression by immunohistochemistry revealed regionally variable viral localization in the brain, with central nervous system infection occurring independently of virus-induced blood-brain barrier disruption or experimental macrophage depletion. Furthermore, CC041 mice developed clinical signs consistent with neurological disease that persisted beyond the period when infectious virus was detected in the brain. Together, these findings establish CC041 mice as a tractable, immunocompetent model for studying chikungunya virus-associated neurological disease following peripheral infection.

## Author summary

Chikungunya virus is a mosquito-borne infection that continues to spread worldwide and can cause long-lasting neurological problems in some individuals. We still do not fully understand how the virus invades the brain or why only certain people develop severe neurological symptoms. A significant challenge has been the lack of a small mammalian model with an intact immune system that develops brain infection via a natural route of exposure. In our study, we identified a mouse strain, CC041, that is uniquely vulnerable to chikungunya virus entering the brain via an inoculation route similar to a mosquito bite. These mice show viral spread throughout the body, including detectable virus in the brain, and clinical signs of neurological disease that persist even after infectious virus is no longer detectable in the brain. By comparing CC041 mice to a mouse strain resistant to brain infection, we found that susceptibility was linked to the virus's ability to spread throughout the body and to the duration of virus circulation in the bloodstream, rather than to differences in virus replication at the infection site. This mouse model provides an essential tool for understanding how chikungunya virus affects the central nervous system.

## Introduction

Chikungunya virus (CHIKV) represents a re-emerging global public health threat. This Old-World alphavirus, transmitted by *Aedes aegypti* and *Aedes albopictus* mosquitoes*,* continues to expand its geographic range and cause large-scale outbreaks across India, Southeast Asia, and the Americas. CHIKV causes millions of infections annually, accounting for an estimated average loss of more than 106,000 disability-adjusted life years (DALYs) every year [1]. As of December 2025, CHIKV has been declared a high-risk threat by the World Health Organization due to frequent outbreaks, including in Guangdong Province, China, and multiple countries in South America [2,3]. Since January 2025, more than 240,000 cases and 90 CHIKV-related deaths have been reported across 16 countries and territories, including a locally-acquired infection in New York, USA, in October 2025 [2,4].

CHIKV infection typically presents as an acute febrile illness with arthralgia and rash. However, neurological complications are being increasingly recognized, with up to 25% of cases presenting with neurological manifestations, such as encephalitis, altered consciousness, seizures, cognitive dysfunction, and motor impairment [5,6]. Infants under one year of age are especially at risk, with an estimated 25–28% of infected children developing neurological symptoms [5,7]. Despite the abundance of documented CHIKV neurological disease cases, the mechanisms underlying CHIKV neuropathogenesis remain poorly understood, limiting the development of effective countermeasures.

Until recently, prevention strategies were limited to vector control and personal protection, as no licensed CHIKV vaccines were available. This status changed in late 2023, when the United States Food and Drug Administration (FDA) approved IXCHIQ, a single-dose live-attenuated vaccine for adults, followed soon after by approvals in Canada and Europe [8]. However, early post-market surveillance identified safety concerns, including neurological and cardiac adverse events in older adults, prompting updated guidance and a temporary pause in vaccine use for specific populations in 2025 [9]. More recently, the approval of VIMKUNYA^TM^, a single-dose virus-like particle-based CHIKV vaccine for individuals aged 12 years and older, represents a significant advance. Regardless, limited understanding of CHIKV neuropathogenesis continues to constrain evaluation of vaccine safety, efficacy, and durability, particularly in populations at risk for neurological disease that are not eligible for vaccination [10,11]. These developments underscore the urgent need for a deeper understanding of CHIKV neuropathogenesis to inform the development of safer, more effective vaccines and therapeutics, particularly given the absence of approved antiviral therapies and the significant risk of severe or chronic disease accompanying the continued geographic expansion of competent mosquito vectors [5,12].

One significant barrier to advancing such interventions is the limited ability to study CHIKV infection within the human central nervous system (CNS), highlighting the need for robust experimental models. Several animal systems, including zebrafish [13,14] and nonhuman primates (NHPs) [15,16], have provided valuable insights into CHIKV pathogenesis, but each model has fundamental limitations. Zebrafish lack a mammalian immune system, limiting their translational relevance, whereas NHPs provide high-fidelity disease modeling but are costly and typically reserved for late-stage therapeutic evaluation. In contrast, mice offer substantial advantages for studying CHIKV neuropathogenesis, including a fully functional mammalian CNS and immune system, extensive availability of species-specific reagents, and tractable genetic tools that facilitate mechanistic inquiry. Young adult (4–6-week-old) mice, which are neurodevelopmentally equivalent to a 1–2-year-old child [17], one of the human age groups most susceptible to CHIKV encephalitis [7], possess ideal immune and neurodevelopmental statuses for modeling CHIKV-induced neurological disease. Developing an immunocompetent young-adult mouse model capable of developing CNS infection after peripheral inoculation is therefore essential to dissect the mechanisms of CHIKV neuroinvasion and neuropathogenesis.

To address this gap, our team leveraged the genetically diverse Collaborative Cross (CC) recombinant inbred panel and identified a novel mouse strain, CC041/TauUncJ (CC041), that is naturally susceptible to CHIKV neuroinvasion after peripheral inoculation as young adults [18,19]. CC041 mice uniquely supported consistent CNS infection following subcutaneous footpad (SQ FP) injection in a CHIKV strain-, but not a CHIKV dose-dependent manner. These mice also demonstrated viral dissemination and signs of neurological disease that persisted even after infectious virus was no longer detectable in the brain, similar to CHIKV neuropathogenic manifestations in humans [7]. These findings establish CC041 mice as a robust, immunocompetent, developmentally appropriate model for studying CHIKV neuroinvasion and neuropathogenesis following a more natural route of infection.

## Results

### CC041 mice are susceptible to CHIKV neuroinvasion in a CHIKV strain-dependent manner

Standard inbred mouse strains, such as C57BL/6J (B6), are typically not susceptible to CHIKV neuroinvasion following peripheral infection after weaning age [20]. Therefore, we turned to the genetically diverse CC recombinant inbred mouse panel to identify alternative susceptible mouse strains. Ten different CC mouse lines were inoculated via SQ FP injection with SL15649, a CHIKV strain previously shown to be neurovirulent in young adult B6 mice following direct intracranial (IC) inoculation [20]. At 5 days post-infection (DPI), brain viral titers varied widely across CC lines, with CC007/Unc (CC007) and CC041 mice demonstrating consistent rates of neuroinvasion (S1A Fig). While neuroinvasion was not observed in a follow-up experiment in CC007 mice (S1B Fig), longitudinal analysis in CC041 mice confirmed detectable virus in the brain as early as 3 DPI, peaking at 5 DPI, and declining below the assay limit of detection (LOD) by 10 DPI (S1C Fig). Based on their reproducible susceptibility to CHIKV neuroinvasion, CC041 mice were selected for further characterization.

**Fig 1:**
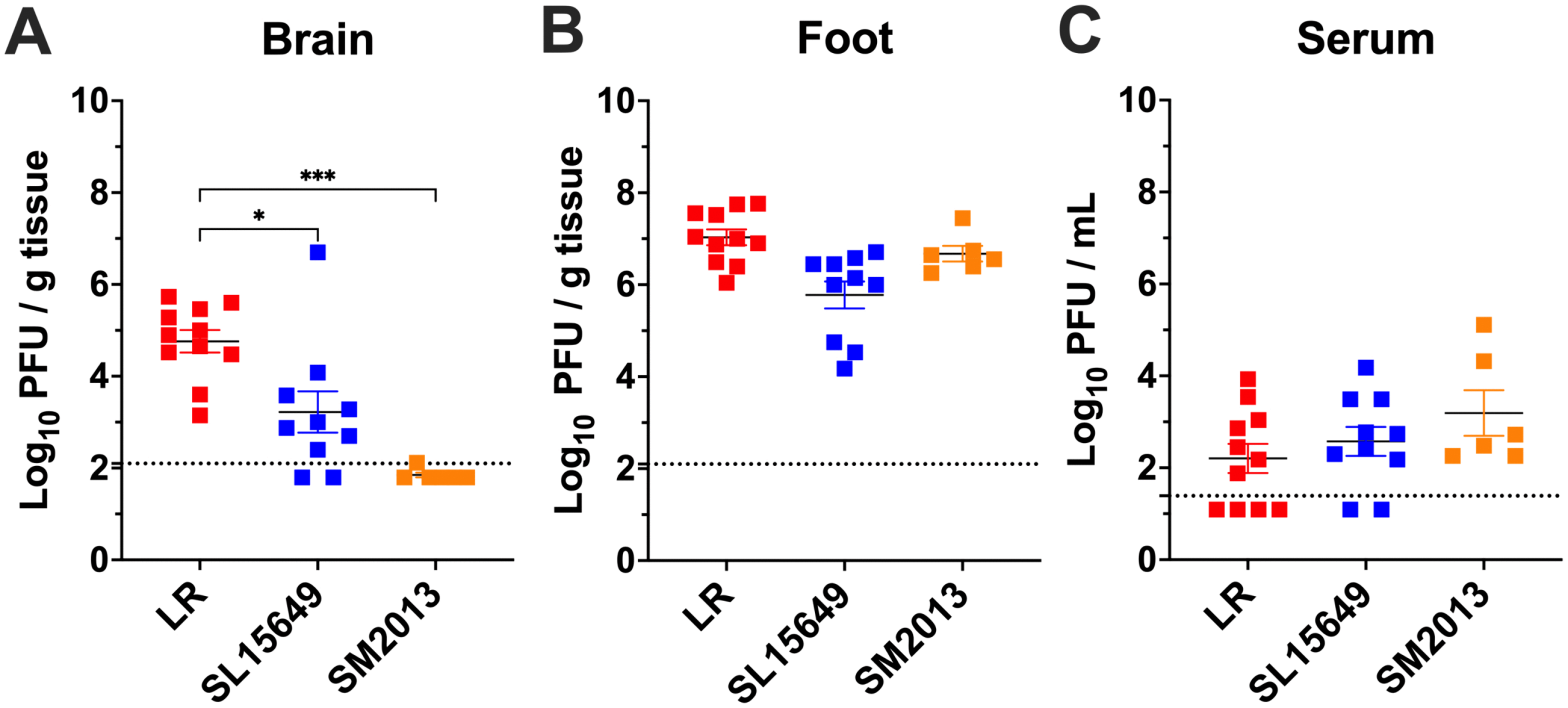
CHIKV titers in CC041 mice peripherally infected with different CHIKV strains. CC041 mice were inoculated SQ via FP with 10^3^ PFU of either CHIKV LR (red symbols), SL15649 (blue symbols), or SM2013 (orange symbols). At 5 DPI, brain **(A)**, ipsilateral foot **(B)**, and serum **(C)** were collected, and viral titers were determined by plaque assay. Assay limit of detection (LOD) is depicted by the horizontal dotted line; all samples that fell below the plaque assay LOD were assigned a value of half of the LOD. * p < 0.05, *** p < 0.001, Kruskal-Wallis test.

We next evaluated whether neuroinvasion in CC041 mice varied by CHIKV strain. Young adult (4–6-week-old) male and female CC041 mice were infected via SQ FP with a physiologically relevant dose of 10^3^ plaque-forming units (PFU) [21] with one of three CHIKV strains: two East/Central/South African (ECSA) lineage strains, La Réunion (LR) and SL15649, and one Asian lineage strain, SM2013. CC041 mice infected with ECSA lineage CHIKV strains exhibited consistent viral titers in the brain at 5 DPI, with CHIKV LR generating the highest titers (Fig 1A). In contrast, CC041 mice infected with the Asian lineage strain SM2013 rarely showed detectable CNS infection. Ipsilateral foot and serum viral titers did not differ significantly across CHIKV strains (Fig 1B and 1C), indicating that the CHIKV strain-specific neuroinvasive capacity is not attributable to differences in peripheral replication or systemic viral load. Because the ECSA-lineage CHIKV strains, LR and SL15649, consistently resulted in neuroinvasion, these two strains were used for subsequent studies. For clarity and consistency, CHIKV LR data are denoted with red or maroon symbols (CC041 mice) and black and gray symbols (B6 mice), whereas CHIKV SL15649 data are denoted by bright blue (CC041 mice) and navy (B6 mice) symbols throughout the figures.

We next assessed whether the dose of CHIKV inoculated in the foot influenced subsequent viral load in the brain. CC041 mice were infected with CHIKV LR or SL15649 at either the physiologically relevant 10^3^ PFU dose as before, or at 10^5^ PFU, a 100-fold higher dose. For CHIKV LR, brain titers at 5 DPI were comparable across doses (S2A Fig), and no dose-dependent differences were observed in foot or serum titers (S2B and S2C Fig). Similar patterns were observed for CHIKV SL15649, although brain titers trended lower in mice infected with the 10^3^ PFU dose (S2D–S2F Fig). Because of these findings, for subsequent studies, infections were performed using the physiologically relevant 10^3^ PFU inoculum dose.

### CHIKV dissemination is widespread in CC041 mice, but not in B6 mice, following SQ FP inoculation

**Fig 2:**
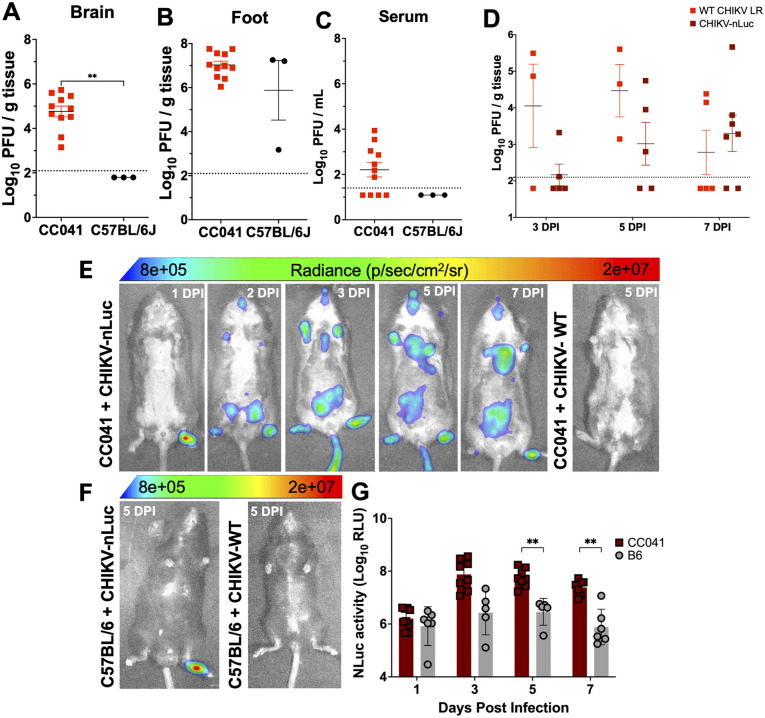
Viral infection dynamics in CC041 and B6 mice following peripheral CHIKV infection. CC041 and C57BL/6J mice were infected SQ via FP with 10^3^ PFU of CHIKV nLuc-expressing CHIKV LR strain (CHIKV-nLuc) or the wild-type (WT) CHIKV LR strain. A-C) Brain **(A)**, ipsilateral foot **(B)**, and serum **(C)** viral titers in CC041 (red squares) and B6 mice (black circles) at 5 DPI. **D)** Viral brain titers of WT CHIKV LR- (red squares) and CHIKV-nLuc (maroon squares)-infected CC041 mice at 3, 5, and 7 DPI. E,F) Representative images of CC041 **(E)** and B6 **(F)** mice imaged via the IVIS spectral imaging system. **G)** Quantification of nLuc signal from 1 to 7 DPI in CC041 (maroon squares) and B6 (gray circles) mouse heads from IVIS spectral images. Assay limit of detection (LOD) is depicted by the horizontal dotted line (A-D); all samples that fell below the plaque assay LOD were assigned a value of half of the LOD. *p < 0.05, **p < 0.01, Multiple Mann-Whitney (A) and Šidák’s multiple comparisons (G) tests.

We next compared the dynamics of CHIKV dissemination and neuroinvasion in susceptible CC041 mice and resistant B6 mice. CC041 mice infected with CHIKV LR exhibited consistent CNS infection at 5 DPI, whereas B6 mice showed no detectable virus in the brain (Fig 2A), despite comparable viral replication in the inoculated foot (Fig 2B). This finding is consistent with previous studies in B6 mice using other CHIKV strains [20]. Although not statistically significant, CHIKV remained detectable in the serum of most CC041 mice at 5 DPI; in contrast, virus in the serum had largely cleared in B6 mice by that time (Fig 2C), suggesting prolonged systemic viral presence in CC041 mice.

To visualize viral dissemination *in vivo*, we employed a recombinant nanoluciferase-expressing CHIKV LR (CHIKV-nLuc) [22]. Brain titers were slightly lower in the CHIKV-nLuc-infected mice than those infected with WT CHIKV LR at 3 DPI but were otherwise comparable through 7 DPI (Fig 2D), validating the reporter virus for imaging. In CC041 mice, *in vivo* imaging system (IVIS) measurements revealed that at 1 DPI, luminescent signal was restricted to the inoculated foot; by 2 DPI, signal appeared at distal anatomical sites consistent with draining inguinal lymph nodes and was observed in the head for the first time (Fig 2E). By 3 and 5 DPI, the CHIKV-nLuc signal was widespread throughout the body before beginning to decline by 7 DPI. In stark contrast, B6 mice rarely demonstrated detectable CHIKV-nLuc signal outside the inoculated foot at any time point (Fig 2F). While whole body imaging precluded precise anatomical localization of signal, particularly in distinguishing signal originating from the brain versus adjacent structures, quantification of cranial radiance revealed significantly higher viral signal in the heads of CC041 mice compared to B6 mice at 5 and 7 DPI (Fig 2G), further supporting the enhanced neuroinvasive susceptibility of CC041 mice.

### Systemic and peripheral viral replication and type I interferon levels differ between CHIKV neuroinvasion-susceptible and neuroinvasion-resistant mouse strains during early infection

**Fig 3:**
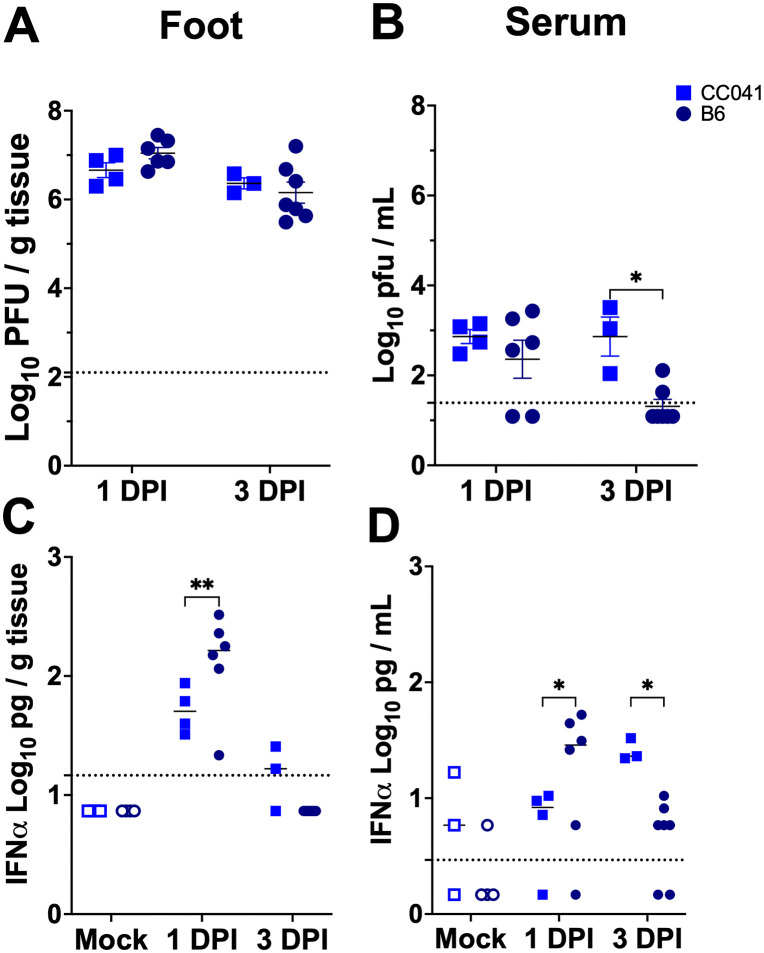
CHIKV titers and IFN-α levels early after CHIKV infection in the periphery of CC041 and B6 mice. CC041 (bright blue squares) and C57BL/6J (navy circles) mice were infected SQ via FP with 10^3^ PFU of CHIKV SL15649. Infectious virus was quantified by plaque assay **(A, B)**, and IFN-α was quantified by Luminex **(C, D)** in clarified left hind foot homogenates and sera. Assay limit of detection (LOD) is depicted by the horizontal dotted line; all samples that fell below the assay LOD were assigned a value of half of the LOD. *p < 0.05, **p < 0.01, Šidák’s multiple comparisons tests.

After determining that neuroinvasion begins as early as 2 DPI in CC041 mice, we next evaluated early peripheral events that may influence susceptibility to CNS infection. CHIKV SL15649 (Fig 3A) and LR (S3A Fig) replication in the inoculated foot was comparable between CC041 and B6 mouse strains at 1 and 3 DPI, indicating that differences in neuroinvasion are not attributable to local replication efficiency. In contrast, CHIKV SL15649 induced significantly higher serum viral loads at 3 DPI in CC041 mice (Fig 3B). Serum viral loads similarly trended higher in CHIKV LR-infected mice at 1 and 3 DPI (S3B Fig), consistent with the prolonged serum viral load observed with CHIKV LR infection at 5 DPI (Fig 2C).

Type I interferons (IFNs), IFN-alpha (IFN-α) and IFN-beta (IFN-β), are essential early antiviral cytokines that limit alphavirus replication and systemic spread by inducing IFN-stimulating genes (ISGs) that establish an antiviral state in infected and neighboring cells [23]. As type I IFN responses help control alphavirus dissemination [24], we quantified IFN-α and IFN-β levels in non-neurologic compartments early after infection. CHIKV-infected B6 mice exhibited significantly higher IFN-α levels in both the ipsilateral foot (Fig 3C, 17-fold increase from baseline to peak levels) and serum (Fig 3D, 26-fold increase from baseline to peak levels) at 1 DPI. In contrast, CC041 mice showed only a sevenfold increase in IFN-α levels in the ipsilateral foot at peak viral load. CC041 mice also had a delay in peak serum IFN-α concentrations, although the magnitude was similar to that in B6 mice (26-fold increase from baseline to peak levels). IFN-β concentrations in the ipsilateral foot followed similar kinetics to IFN-α in both CC041 and B6 mice (S3C Fig); however, IFN-β levels in both strains were largely undetectable in serum (S3D Fig). These measurements demonstrate that peripheral type I IFN response dynamics, particularly IFN-α, differ in magnitude and timing between CC041 and B6 mice, with the neuroinvasion-resistant B6 mice exhibiting higher IFN levels at the site of initial CHIKV replication.

To further compare the peripheral and systemic immune response to CHIKV infection between CC041 and B6 mice, we evaluated cytokine and chemokine levels in the ipsilateral foot and serum by qPCR and Luminex. Baseline expression of *Infa4, Infb1, Ccl2, Il10, Tnf, Ifng,* and *Il6* in the foot did not signifcantly differ between mock-infected CC041 and B6 mice (S1 Table). Following infection with CHIKV LR, expression of all genes increased in both mouse strains (S3E Fig), with B6 mice demonstrating signficantly upregulated *Tnf* expression at 1DPI and CC041 mice demonstrating significantly upregulated *Il6* expression at 3 DPI. While expression of *Ifna4*, *Ifnb1*, and *Il6* returned to baseline levels by 7–14 DPI, *Ccl2, Il10, Tnf,* and *Ifng* expression remained upregulated at 14 DPI.

Quantification of cytokines and chemokines in the foot and serum up to 5 days following CHIKV LR infection by Luminex (S4 Fig) revealed increased levels of several of the 23 analytes measured. Increased analytes included IL-4, IL-5, IL-10, IL-12p40, CCL4/MIP-1β, CXCL1/KC, and TNF-α in the ipsilateral foot alone and CCL2/MCP-1, CCL3/MIP-1α, CCL5/RANTES, and G-CSF in both the foot and serum. Although a few analytes were significantly higher in mock-infected B6 mice compared to CC041 mice, following CHIKV infection, only limited strain-specific differences were observed. Specifically, B6 mice exhibited higher levels of IL-1β and CCL5/RANTES in the foot at 1 DPI and CCL11/Eotaxin in the foot at all time points, whereas CC041 mice demonstrated significantly higher G-CSF levels in the foot at 5 DPI. Collectively, these findings indicate that CC041 and B6 mice mount broadly comparable local and systemic proinflammatory cytokine and chemokine responses following CHIKV infection.

### Evaluation of CHIKV localization and entry into the CNS

**Fig 4:**
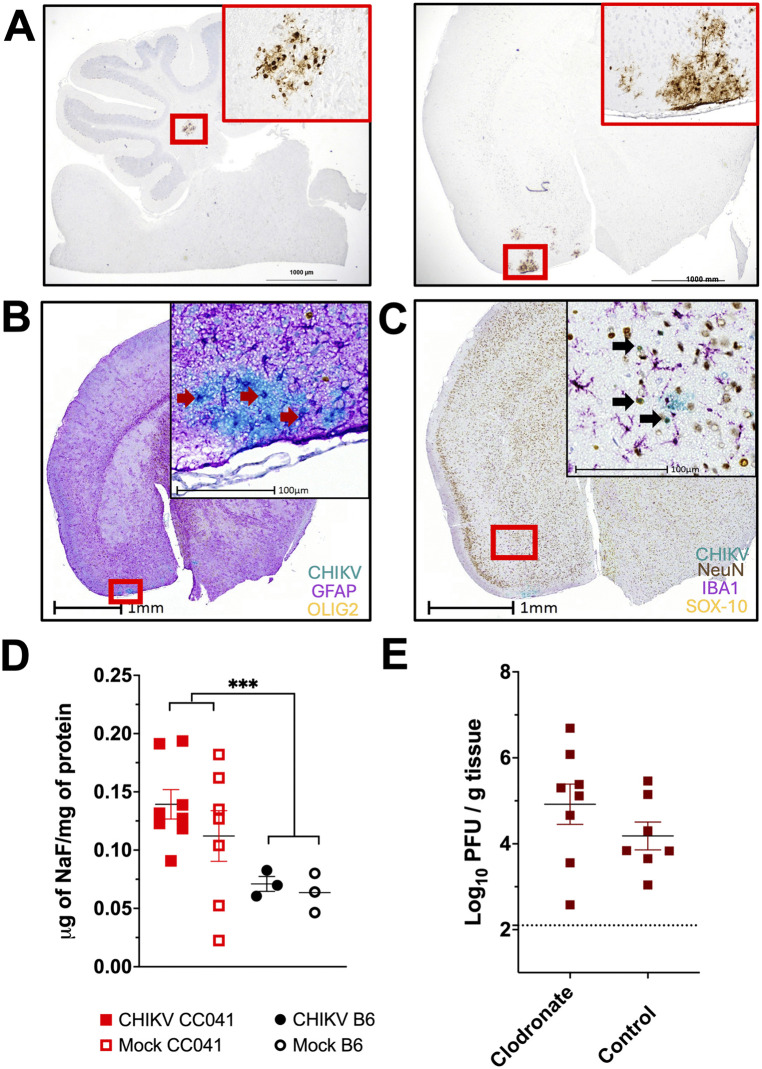
CHIKV localization and entry into brains of CC041 mice following peripheral infection. **A)** CC041 mouse brain sections of cerebellum (left image) and cerebral cortex (right image) at 5 DPI following 10^3^ PFU CHIKV LR infection SQ via FP showing positive CHIKV antigen labeling (brown chromogen, insets) by IHC. **B)** Cerebral brain section of a CHIKV-LR-infected CC041 mouse at 5 DPI labelled with 3plex-chromogenic IHC showing CHIKV antigen-positive cells (teal chromogen) colocalizing (dark blue color denoted by red arrows) with astrocyte marker (GFAP) identified by purple chromogen. Oligodendrocytes (OLIG2) are denoted with yellow chromogen. **C)** Cerebral brain section of a CHIKV-LR-infected CC041 mouse at 5 DPI labelled with 4plex-chromogenic IHC showing CHIKV antigen-positive cells (teal chromogen) colocalizing (green color denoted by black arrows) with oligodendrocyte marker (SOX-10) identified by yellow chromogen. Neurons (NeuN) are identified by brown chromogen, and microglia (IBA1) are identified by purple chromogen. **D)** CC041 (red symbols) and B6 (black symbols) mice were infected with 10^3^ PFU of CHIKV LR (closed symbols) or mock-infected with PBS (open symbols) and euthanized at 5 DPI following an intraperitoneal injection of 100 mg/mL of sodium fluorescein (NaF). NaF and total protein were quantified in 20% w/v brain homogenates immediately following necropsy. **E)** CHIKV titers at 5 DPI in brains of CC041 mice who received clodronate liposomes to deplete macrophages (“Clodronate”) or control liposomes (“Control”) 2 days prior to infection with 10^3^ PFU CHIKV-nLuc (maroon squares) via SQ FP inoculation. Plaque assay limit of detection (LOD) is depicted by the horizontal dotted line. ***p < 0.001, Mann-Whitney test (D).

We next returned our attention to the CNS and evaluated the location(s) where CHIKV infects the CC041 mouse brain by labeling formalin-fixed, paraffin-embedded brain sections for CHIKV antigen by immunohistochemistry (IHC). Even at 5 DPI, when brain viral titers peak in CC041 mice (S1C Fig), CHIKV antigen (brown chromogen) was detected only sporadically in brain sections. Additionally, when present, CHIKV antigen labeling varied across brain regions. Regions where CHIKV antigen was detected included the cerebellar nuclei and the cerebral cortex, where antigen-positive cells extended from the meninges into the brain parenchyma (Fig 4A). This nonspecific pattern of CHIKV localization is consistent with hematogenous entry of virus into the CNS, as observed in fatal human CHIKV cases and zebrafish models [14,25].

**Fig 5:**
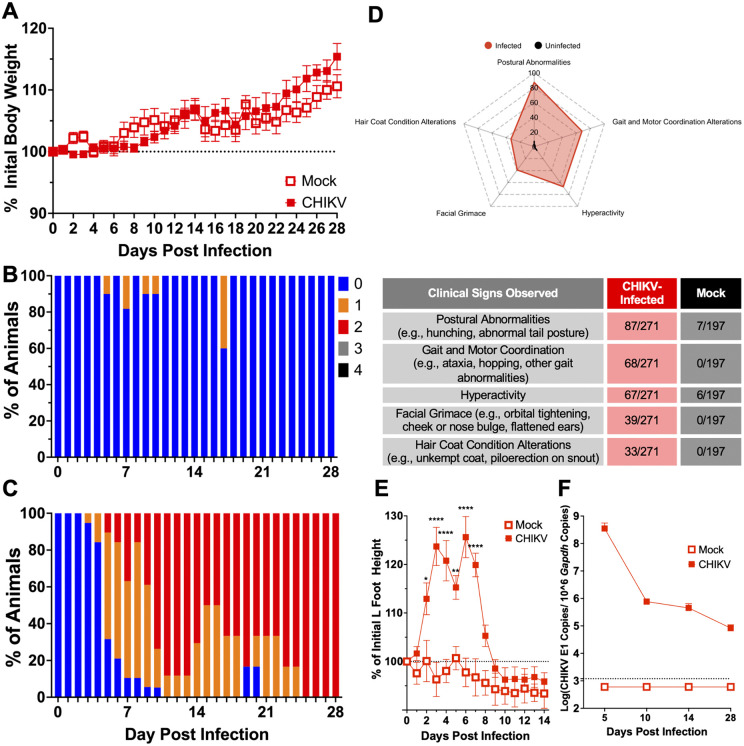
Clinical disease evaluation in CHIKV-infected CC041 mice. CC041 mice were infected with 10^3^ PFU CHIKV LR or mock-infected with PBS SQ via FP. **A)** Mock-infected (open symbols, n = 25) and CHIKV-infected (closed symbols, n = 49) mice were weighed daily. Clinical scores in mock-infected (**B**, n = 9) and CHIKV-infected (**C**, n = 13) CC041 mice were assigned to each mouse daily as follows: 0 = clinically normal; 1 = mild abnormal gait and tail posture; 2 = hunched posture, abnormal gait, unkempt hair coat; 3 = marked hunched posture, and decreased ambulation (humane endpoint); 4 = moribund/dead/euthanized. During daily clinical observations, qualitative descriptors were recorded, including facial grimace, hyperactivity, abnormal behaviors (e.g., digging, excessive grooming), altered gait or posture (e.g., wide-based gait, wide rear-limb posture), and abnormal tail carriage (e.g., stiff-tail, tail-flagging). **D)** Spider plot illustrating the relative frequency of qualitative descriptors observed in CHIKV LR- (red shading) and mock-infected (black shading) CC041 mice. **E)** Foot measurements were measured daily in sedated mock-infected (open symbols, n = 5) or CHIKV LR-infected (closed symbols, n = 6) CC041 mice. **F)** Viral RNA was quantified by qPCR in mock-infected (open symbols, n = 1-6 mice/timepoint) or CHIKV LR-infected (closed symbols, n = 2-6 mice/timepoint) CC041 left brain hemispheres. Data are presented as the mean SEM; in (A) and (E), the horizontal dotted line represents the 0 DPI value to which values at all subsequent timepoints were normalized; in (F), the assay limit of detection (LOD) is depicted by the horizontal dotted line; all samples that fell below the plaque assay LOD were assigned a value of half of the LOD; * p < 0.05, ** p < 0.01, **** p < 0.0001, Šidák’s multiple comparisons test.

We next performed multiplex-chromogenic IHC to determine which resident CNS cells (e.g., neurons, astrocytes, oligodendrocytes, and microglia) were targeted by CHIKV (Fig 4B and 4C). CHIKV antigen, labelled with teal chromogen, frequently colocalized with GFAP+ astrocytes labeled with purple chromogen (colocalized areas seen as dark blue and denoted with red arrows, Fig 4B). CHIKV was also found to occasionally colocalize with the oligodendrocyte marker labeled with a yellow chromogen (colocalized areas shown in green and denoted with black arrows, Fig 4C) but was not detected in NeuN+ neurons (Fig 5C, brown chromogen). CHIKV was also not detected in IBA1+ microglia (Fig 5C, purple chromogen), in line with previous reports that microglia are not permissive or only poorly permissive to CHIKV replication [26]. After quantifying comparable numbers of astrocytes and oligodendrocytes in brain sections, we found 0.66% of astrocytes were infected with CHIKV compared to 0.093% of oligodendrocytes, indicating that astrocytes were infected approximately 7X more frequently than oligodendrocytes. These findings suggest astrocytes are the primary resident CNS cellular target of CHIKV in CC041 mice, consistent with previous reports [27].

Because several viruses (e.g., human immunodeficiency virus, Japanese encephalitis virus, and Zika virus [28–30]) increase blood-brain barrier (BBB) permeability to facilitate CNS entry, we assessed BBB integrity by measuring sodium fluorescein (NaF) accumulation in brain homogenates of neuroinvasion-susceptible CC041 mice and neuroinvasion-resistant B6 mice. NaF levels did not differ significantly between CHIKV-infected and mock-infected mice in either CC041 or B6 strains at 2 DPI (S5A Fig) or 5 DPI (Fig 4D). However, CC041 mice exhibited significantly higher baseline NaF brain accumulation than B6 mice, regardless of infection status, suggesting that CC041 mice possess an inherently more permeable BBB.

C-C motif chemokine ligand 2 (CCL2) signaling has also been found to promote the transmigration of CHIKV-infected monocytes across the BBB in fatal human CHIKV cases and *in vitro* [25], suggesting that CHIKV may disseminate throughout the body via infected circulating monocytes and enter the CNS via a “Trojan horse” mechanism. Therefore, we next assessed macrophage involvement in CHIKV neuroinvasion in CC041 mice by depleting monocytes and macrophages with clodronate liposomes or mock-depleting with control liposomes two days before infection with CHIKV LR (S2 and S3 Tables). Monocyte and macrophage depletion did not significantly alter brain titers at 5 DPI (Fig 4E), nor did it affect clinical scores compared to mice administered control liposomes (S5B and S5C Fig), indicating that macrophages are not required for CHIKV entry into the CNS in CC041 mice.

### CHIKV-infected CC041 mice develop persistent clinical signs consistent with neurological dysfunction

To evaluate the clinical impact of peripheral CHIKV infection in CC041 mice, animals were assessed for weight loss and clinical disease through 28 DPI using a clinical scoring system adapted from that for IC CHIKV infection in B6 mice [20]. No weight loss was observed in mock-infected or CHIKV-infected mice at any time during the period of observation (Fig 5A). Mock-infected CC041 mice remained clinically normal throughout the study, rarely scoring above a ‘0’ (i.e., no clinical signs of disease) (Fig 5B), whereas CHIKV-infected CC041 mice exhibited progressively elevated clinical scores. Although no mortality was observed, approximately half of the infected CC041 mice reached a clinical score of “2” by 10 DPI, and all CHIKV-infected CC041 mice reached this score between 25 and 28 DPI (Fig 5C). In contrast, B6 mice, whether mock-infected or CHIKV infected, showed comparable weight gain (S6A Fig) and remained largely clinically normal through 14 DPI, with only occasional sporadic clinical scores of “1” observed (S6B and S6C Fig).

Several clinical phenotypes displayed by CHIKV-infected CC041 mice (S1 Video) but not observed in mock-infected mice (S2 Video) were consistent with neurological dysfunction, including postural abnormalities, gait abnormalities, hyperactivity, facial grimace, and hair coat condition alterations (Fig 5D). Abnormal tail posture (S7 Fig), characterized by rigidity or a persistent upward curvature, was an especially notable feature frequently observed in CHIKV-infected CC041 mice. Although the specific neurological phenotypes varied among mice, CHIKV-infected animals consistently exhibited these signs until the study concluded at 28 DPI. In contrast, neurological phenotypes in B6 mice, whether CHIKV-infected or mock-infected, were limited to rare, sporadic observations (S6D Fig). These results demonstrate that young-adult CC041 mice develop prolonged clinical signs consistent with neurological disease following peripheral CHIKV exposure.

To assess whether development of the clinical signs observed in CHIKV-infected CC041 mice could be attributed to local inflammation at the site of inoculation, footpad swelling was measured. We focused on CC041 mice, as swelling kinetics following footpad injections in B6 mice following CHIKV infection are already well characterized [31,32]. Foot size increased significantly in CHIKV-infected mice relative to mock-infected mice by 2 DPI, several days before increased clinical scores were observed, and returned to baseline by 9 DPI (Fig 5E). Furthermore, clinical signs consistent with neurological dysfunction persisted well beyond the resolution of measurable swelling, suggesting that local swelling at the site of inoculation alone does not account for the observed clinical signs in CC041 mice.

To determine if persistent or reactivated virus is associated with the development of clinical signs, we quantified both infectious virus by plaque assay and viral RNA by qPCR. Infectious virus was undetectable at 10, 14, and 28 DPI (S8 Fig); however, detectable viral RNA persisted in the brain through 28 DPI (Fig 5F). These data suggest that persistent viral RNA could be contributing to the development of clinical signs consistent with neurological dysfunction.

## Discussion

In this study, we identified young adult CC041 mice as a unique immunocompetent small mammalian model that supports CHIKV neuroinvasion following physiologically relevant peripheral infection. The CC041 mouse model addresses a longstanding limitation in the field, as CNS involvement in CHIKV infection has been challenging to study in systems that preserve immune competence, neurodevelopmental maturity, and natural routes of exposure. Although prior work has established that mice are susceptible to CHIKV infection in the CNS [27,33,34], susceptibility is dependent on host age and viral strain [20], and most observations derive from experimental systems that do not reflect typical routes of human exposure.

Historically, studies of CHIKV neuropathogenesis have relied on neonatal mice, severely immunocompromised mice, or direct IC inoculation to achieve neurological CHIKV infection, because post-weanling-age, immunocompetent, standard inbred mouse strains do not develop CNS infection following peripheral (e.g., footpad or subcutaneous) inoculation. However, each of these approaches has significant limitations. Neonatal mice, while permissive to CNS infection by CHIKV, are neurodevelopmentally equivalent to a third-trimester fetus or a newborn human [17]. While helpful for studying susceptibility to neurological infection, their lack of neurodevelopmental translatability makes them poorly suited for modeling neuroinvasion, age-appropriate immune responses, or neurological disease progression. Immunocompromised mouse strains tend to develop more robust CNS infection but distort key aspects of host-virus interactions that shape neuropathogenesis in immunocompetent hosts. Direct IC inoculation allows controlled CNS infection but artificially bypasses peripheral viral replication and consequent local and systemic host inflammatory responses, hematogenous dissemination, and natural barriers to neuroinvasion. *In vitro* systems, such as human microglial cell lines and neurospheres, provide valuable insights into cellular responses and viral replication dynamics but lack the complex interactions of multiple cell types, immune responses, and the BBB, all of which are critical for understanding CHIKV neuroinvasion and disease progression in humans [35–37]. While these models have provided valuable insights into CHIKV pathogenesis, they cannot fully recapitulate the systemic viral dissemination, immune-mediated pathology, or the natural route of infection, limiting their ability to predict clinical outcomes or evaluate therapeutics effectively. Given the frequency of neurological complications in infants and young children infected with CHIKV, evaluating novel models that preserve intact antiviral immunity, age-appropriate neurodevelopment, and peripheral routes of inoculation are critical for understanding how viral and host factors contribute to neuroinvasion and disease.

The Collaborative Cross (CC) is a multi-parent recombinant inbred mouse reference population designed to capture genetic diversity comparable to that of the human population, providing a reproducible platform for both novel phenotype discovery and high-resolution genetic mapping [38,39]. As several genetic factors have been associated with susceptibility to CHIKV-induced disease in humans, including genetic polymorphisms in human leukocyte antigens (HLAs), pathogen-recognition receptors (PRRs), and cytokines [40–45], the ability to evaluate how host genetic variation influences disease manifestations is critical for understanding pathogenesis and identifying mechanisms underlying differential disease outcomes. For our CHIKV neuropathogenesis studies, the CC framework enabled identification of a permissive yet immunocompetent host background that was not represented by standard inbred mouse strains, such as B6. This finding highlights the ability of CC strains to reveal extreme responder phenotypes and supports expanded screening of the remaining available CC strains to facilitate downstream genetic dissection.

CC041 mice have been previously used to study complex genetic traits, with their resistance to cocaine-induced behavioral sensitization being particularly well documented [39,46–48]. However, to our knowledge, CC041 mice have not been reported to be uniquely sensitive to viral infection. While defining the specific genetic determinants that distinguish CC041 mice from neuroinvasion-resistant B6 mice will require dedicated genetic mapping studies, the strain divergence observed here underscores the central value of the CC framework for uncovering host factors that modulate viral neuroinvasion. The CC041 mouse model, therefore, provides a tractable platform for dissecting mechanisms that have remained largely inaccessible in human populations and other model systems.

Our findings support our hypothesis that permissive host genetics, in combination with viral strain-specific properties, govern susceptibility to CHIKV neuroinvasion following peripheral infection, with CC041 mice demonstrating CNS infection following SQ FP infection with CHIKV strains belonging to the ECSA, but not Asian, phylogenetic clade. Viral lineage has also been implicated in human neurological CHIKV disease, with encephalitis reported in association with both Asian and ECSA lineage strains, but not West African lineage strains, supporting the inclusion of multiple CHIKV strains in experimental models of neuroinvasion [49]. In contrast to the CHIKV strain-dependent susceptibility observed in CC041 mice, the widely used B6 mouse strain is resistant to CNS infection across multiple CHIKV strains following physiologically relevant doses (i.e., 10^3^ PFU) and routes of infection [20,50,51]. Notably, increasing the inoculum dose by 100-fold did not significantly increase brain viral titers for CHIKV LR in CC041 mice, suggesting that once systemic infection occurs, CNS seeding may be constrained by host barriers or innate immune control that cannot be overcome by additional virus input. This observation further supports the concept that neuroinvasion in CC041 mice reflects intrinsic host susceptibility rather than simple dose-dependent spillover.

A defining difference observed between infected CC041 and B6 mice was the extent and duration of systemic viral burden following peripheral infection. Although viral replication at the inoculation site was comparable between mouse strains, CC041 mice maintained significantly higher serum viral loads and exhibited widespread viral dissemination beginning as early as 2 DPI. In contrast, B6 mice effectively restricted infection to the inoculation site, showing minimal viral dissemination. Sustained serum viral load and broad dissemination are well-recognized risk factors for neuroinvasion in alphavirus infections [33,24], and the elevated CNS susceptibility observed in CC041 mice is consistent with this infection profile.

Type I IFN is critical for early alphavirus control, inducing antiviral effectors such as IFIT1, which inhibits translation of alphavirus genomic RNA [52], IFITM3, which restricts viral fusion and entry [53], and PKR, which suppresses viral protein synthesis. During the 2005–2006 CHIKV outbreak in La Réunion, infected patients exhibited serum IFN-α concentrations that were, on average, greater than twofold higher than those of uninfected controls [54,55]. In this context, the sevenfold and twenty-sixfold increase in IFN-α levels relative to baseline observed in CC041 and B6 mice, respectively, suggest that IFN-α responses measured here fall within a physiologically meaningful range.

Prior studies in Sindbis virus (SINV) [56] and Venezuelan equine encephalitis virus (VEEV) [57] mouse models have demonstrated that failure to activate IFN signaling early in infection permits uncontrolled systemic viral replication and markedly increases CNS invasion [33]. Consistent with this framework, differences in systemic viral burden between CC041 and B6 mice found in our study appear to be driven by the timing and magnitude of the type I IFN response. B6 mice mounted a rapid IFN-α and IFN-β response early after CHIKV infection (1–3 DPI), whereas CC041 mice exhibited delayed induction and lower peak IFN-α and IFN-β levels at the site of initial viral replication. Taken together, these findings support a mechanistic model in which delayed type I IFN induction in CC041 mice leads to less regulation of early viral replication, resulting in prolonged serum viral titers and extensive dissemination, thereby increasing the likelihood of CNS seeding [33]. Conversely, the rapid IFN-α and IFN-β response in B6 mice may restrict viral amplification early enough to prevent systemic spread and subsequent neuroinvasion [24]. Although correlative, these results emphasize that the timing of innate immune activation, rather than its magnitude alone, may be a critical determinant of susceptibility to CHIKV neuroinvasion.

The adaptive immune system plays a critical role in viral clearance and disease pathogenesis in both CHIKV and other encephalitic alphaviruses [20,58]. Previous studies characterizing immune cell populations across available CC strains at baseline have demonstrated that CC041 mice possess major adaptive immune cell populations, including CD4+ T cells, CD8+ T cells, and B cells, and antibodies [59,60]. Although an in-depth evaluation of the adaptive immune response was beyond the scope of this study, we observed a >94-fold increase in *Ifng* expression in CC041 mouse feet by 7 DPI, suggesting activation of antiviral immune responses involving NK and/or T cells. Additionally, the increased expression and protein production of multiple proinflammatory cytokines and chemokines in the inoculated feet of CC041 mice, at levels comparable to those observed in B6 mice, indicate that CC041 mice are capable of mounting a multifaceted immune response to CHIKV infection similar to that of standard immunocompetent inbred mouse strains. Nevertheless, future studies characterizing the adaptive immune response to CHIKV infection in CC041 mice and determining whether and how that response differs from that in neuroinvasive-resistant strains, such as B6 mice, are warranted.

Viruses can access the CNS through multiple routes, including hematogenous spread across the BBB (e.g., Epstein-Barr virus, hepatitis C virus, West Nile virus) [61–63], axonal transport along peripheral nerves (e.g., poliovirus, rabies virus, alphaherpesviruses) [64–66], and leukocyte-mediated “Trojan horse” mechanisms (e.g., human immunodeficiency virus, human polyomavirus 2) [67,68]. Recent studies in humans have demonstrated CHIKV-associated perivascular and meningeal inflammation, with additional *in vitro* human BBB models supporting CCL-2-dependent transmigration of CHIKV-infected monocytes across the endothelium [25]. To define how CHIKV gains access to the CNS in CC041 mice, we evaluated virus localization, BBB integrity, and myeloid cell involvement during CHIKV infection. While not conclusive, several lines of evidence from our studies support hematogenous dissemination as the predominant route of CNS entry in the CC041 model. First, CHIKV antigen was detected sporadically across multiple brain regions, including the cerebral cortex and cerebellar nuclei, with antigen-positive cells extending from the meninges into the parenchyma. This distribution argues against entry via discrete neural pathways and instead supports vascular or meningeal access, consistent with observations from fatal human CHIKV cases and zebrafish models of CHIKV [14,25]. Second, despite consistent CNS infection in CC041 mice, CHIKV inoculation via SQ FP did not result in detectable increases in bulk BBB permeability, as measured by NaF accumulation. However, CC041 mice exhibited modestly higher baseline BBB permeability than B6 mice regardless of infection status. These inherent mouse strain-specific differences may lower the threshold for viral entry during sustained viremia, even in the absence of overt BBB disruption. Third, depletion of circulating monocytes and macrophages did not reduce brain CHIKV titers. Studies in zebrafish infected with CHIKV also showed that macrophages are not productively infected and depletion does not delay CNS invasion [14]. Together, these findings argue against a leukocyte-mediated “Trojan horse” mechanism of CNS infection in CC041 mice.

Although CHIKV did not increase BBB permeability in CC041 mice, the virus may still access the CNS through alternative mechanisms, such as endothelial cell infection or transcytosis across the BBB. Other alphaviruses, including VEEV, can traverse the BBB via caveolin-1-dependent transcytosis before detectable barrier disruption [69], and CHIKV LR can infect human brain microvascular endothelial cells *in vitro* [70]. Definitive resolution of these potential mechanisms will require endothelial-targeted *in vivo* analyses and ultrastructural imaging. Future studies that experimentally modulate BBB stability (e.g., pharmacologically enhancing tight junction integrity) may be particularly informative for determining whether baseline barrier properties influence susceptibility to CHIKV neuroinvasion.

CHIKV-infected CC041 mice developed clinical signs consistent with neurological disease, including gait abnormalities, ataxia, hunching, abnormal tail posture, and pronounced hyperactivity, that persisted well after infectious virus was cleared from the CNS. These phenotypes mirror neurological sequelae reported in human pediatric CHIKV survivors [71] and in other alphavirus mouse models [72], including the persistence of behavioral and motor abnormalities beyond the CNS viral replication period [73]. Importantly, our data suggest that these clinical signs are not solely attributable to local inflammation at the site of inoculation. Significant footpad swelling in CC041 mice was first noted at 2 DPI, when all mice were clinically normal, and resolved by 9 DPI, whereas clinical signs persisted through 28 DPI. In addition, infection of B6 mice, which do not develop CNS infection following peripheral inoculation, resulted in only infrequent clinical findings that were not clearly associated with infection. Together, these findings support a neurological basis for the observed phenotype in CC041 mice. However, a partial contribution from peripheral inflammation cannot be excluded, as expression of several proinflammatory genes in the ipsilateral foot remained elevated at 14 DPI. Therefore, mechanical (e.g., von Frey) and thermal (e.g., hot plate) nociception assays [74] in the ipsilateral and contralateral feet of CHIKV-infected mice will be evaluated in future studies to determine the extent to which foot pain and inflammation contribute to the observed clinical signs.

The persistence of neurological signs after viral clearance suggests that post-acute mechanisms, such as sustained neuroinflammation or neural circuit dysfunction, may contribute to long-term disease. The continued presence of viral RNA, even in the absence of active infectious virus production, requires continuous immune surveillance and control in the brain [75–78]. The effects of this ongoing local immune presence, such as low-level inflammation or changes to neuronal function and signaling, could be contributing to the persistent clinical signs observed in CC041 mice [72, 79,80]. Neuroimmune interactions during and after viral clearance can induce alterations in CNS physiology, potentially driving these sequelae [15,81,26]. Comprehensive behavioral and neuropathological analyses will be necessary to define the nature, mechanisms, and chronicity of these deficits.

Our study has several limitations that guide future work. CNS viral titers in CC041 mice were lower than those achieved with mouse-adapted encephalitic alphaviruses, such as SINV, likely limiting sensitivity for antigen detection and constraining definitive cell-type tropism analyses [20,82–84]. In addition, BBB assessment with NaF at peak infection did not reveal changes in overall permeability; however, it may not have detected transient or regional barrier alterations or distinguished endothelial infection from transcytosis-based entry mechanisms. Although macrophage depletion did not reduce brain titers, further characterization of affected myeloid subsets and trafficking will strengthen conclusions about leukocyte-mediated contributions to CHIKV infection in the CNS. The spinal cord was not evaluated in this study, as the primary objective was to characterize neuroinvasion and neuropathology in the brain. However, CHIKV and other encephalitic alphaviruses can cause widespread CNS infection, including involvement of the spinal cord. For example, SINV infects motor neurons within the anterior horn of the spinal cord [83,85], while VEEV infection in mice results in spinal cord inflammation and white matter demyelination [86]. Therefore, determining whether spinal cord pathology contributes to the motor coordination-related phenotypes observed during CHIKV infection represents an important direction for future studies. Finally, while CC041 mice displayed persistent neurological signs after clearance of infectious virus, comprehensive behavioral testing and longitudinal evaluation of neuropathological changes will be required to define the mechanisms and chronicity of post-acute deficits. Future studies combining endothelial-targeted viral localization, ultrastructural imaging, and experimental modulation of BBB stability, together with standardized behavioral and neuropathological assessments, will clarify how host susceptibility permits CNS entry and drives long-term neurological outcomes in the CC041 mouse model.

In summary, CC041 mice represent the first immunocompetent, developmentally appropriate small mammalian model in which CHIKV consistently invades the CNS following a physiologically relevant peripheral infection route and dose. This model recapitulates key features of human pediatric CHIKV disease, including systemic dissemination, CNS infection, and persistent neurological abnormalities, and provides a tractable platform for elucidating the mechanisms of CHIKV neuroinvasion. As CHIKV continues to expand into previously unaffected geographic regions, disproportionately impacting young children, the CC041 model offers a powerful and urgently needed tool to accelerate the development of vaccines, therapeutics, and biomarkers that prevent or mitigate CHIKV-associated neurological disease.

## Materials and methods

### Ethics statement

All procedures were conducted under an animal use protocol (1865MU) approved by the Institutional Animal Care and Use Committee at Texas Biomedical Research Institute, an AAALAC International-accredited institution. All procedures were performed in accordance with the 8^th^ edition of the *Guide for the Care and Use of Laboratory Animals* and complied with institutional and federal regulations. All work conducted with live CHIKV was performed under Biosafety Level 3 (BSL3) containment at Texas Biomedical Research Institute in accordance with the guidelines outlined in the 6^th^ edition of the *Biosafety in Microbiological and Biomedical Laboratories (BMBL)*. Euthanasia was performed in accordance with the 2020 edition of the AVMA Guidelines for the Euthanasia of Animals.

### Cell and virus stocks

CHIKV stocks were made from infectious clones SM2013 (GenBank: MT228631.1) and SL15649 (GenBank: GU189061.1) provided by the Heise laboratory (University of North Carolina at Chapel Hill). The infectious clone of La Réunion (LR) strain (GenBank: DQ443544.2) was generated by Scott Weaver’s laboratory at the University of Texas Medical Branch. The nanoluciferase (nLuc)-expressing CHIKV LR strain reporter virus (CHIKV-nLuc) was generated by William Klimstra’s Laboratory (University of Pittsburgh) [22]. Virus stocks were grown as previously described [20]. Briefly, RNA was electroporated into BHK-21 cells (ATCC stock CCL-10) and supernatant was clarified and collected at 36 hours post-electroporation. Viral stock titers were established by plaque assay on Vero-81 cells (ATCC stock CCL-81).

### Mice

C57BL/6J (B6) mice were originally obtained from Jackson Laboratories, and CC041/TauUncJ (CC041) mice were obtained from the University of North Carolina System Genetics Core Facility. Experimental mice were bred in-house at Texas Biomedical Research Institute. Mice were housed in an ABSL2 facility under a 12:12-hour light-dark cycle in autoclaved cages containing autoclaved Teklad 7097A bedding, sterile Teklad 2920X chow, autoclaved water, and standard environmental enrichment. At least 24 hours prior to infection, mice were transferred to the Animal Biosafety Level 3 (ABSL3) facility to allow for acclimation. Both male and female mice were included in all experiments, with each sex represented at every collection time point. Mice were randomly assigned to experimental groups.

### Mouse infections and clinical monitoring

Mice were infected at 4–6 weeks of age under light isoflurane anesthesia. Infections were performed by subcutaneous injection into the left hind footpad using a 29-gauge insulin syringe, delivering 10 μL PBS containing 10^3^ plaque-forming units (PFU) of CHIKV. Mock-infected controls received 10 μL sterile PBS.

Mice were weighed and clinically evaluated daily by two independent observers blinded to infection status. Clinical scoring was performed using a system adapted from Anderson et al. [20] and expanded to capture a broader range of neurological and behavioral abnormalities. Scores were assigned to each mouse daily as follows: 0 = clinically normal; 1 = mild abnormal gait and tail posture; 2 = hunched posture, abnormal gait, unkempt hair coat; 3 = marked hunched posture, and decreased ambulation (humane endpoint); 4 = moribund/dead/euthanized. Observers additionally recorded qualitative descriptors including facial grimace, hyperactivity, abnormal behaviors (e.g., digging, excessive grooming), altered gait or posture (e.g., wide-based gait, wide rear-limb posture), and abnormal tail carriage (e.g., stiff-tail, tail-flagging). A clinical score of 3 or ≥30% weight loss constituted a humane endpoint, though no animals reached a humane endpoint in any experiment.

Rear footpad swelling was assessed longitudinally using digital calipers. Mice were anesthetized with continuous-flow isoflurane, and rear paw measurements were taken daily while the animals remained under anesthesia via a nose-cone. For each paw, footpad height was measured from the dorsal surface to the plantar surface, and footpad width was measured from the medial to lateral edge at the widest point just above the phalanges. Measurements were collected by a single investigator for both the ipsilateral and contralateral rear paws, with care taken to maintain consistent paw positioning, caliper placement, and pressure across timepoints. These measurements were used to quantify changes in footpad swelling following subcutaneous footpad inoculation.

### Tissue collection and processing

Mice were euthanized by isoflurane overdose. Blood was collected by cardiocentesis into serum separator tubes and centrifuged to remove red blood cells and isolate serum. Mice were transcardially perfused with 15 mL ice-cold PBS to remove circulating blood. Brains were harvested and hemisected sagitally. The right hemisphere was fixed in 4% paraformaldehyde (PFA) for 24 hours at 4°C, washed, and stored in 1X PBS at 4°C. The left brain hemisphere and left hind footpad were flash-frozen on dry ice and stored at -80 °C until processing.

### Infectious virus quantification

Infectious virus in brain, left hind foot, and serum was quantified by plaque assay as previously described [20]. Briefly, brains and feet were homogenized for 2 min to 20% w/v in ice-cold PBS and clarified by centrifugation at 10,000 RPM for 15 min. Homogenates and serum were diluted 10^-1^ to 10^-6^ for plaque assays. Two hundred microliters of each dilution were applied in duplicate to confluent Vero-81 (ATCC CCL-81) monolayers in 12-well plates. Following 1 hour incubation at 37°C with 5% CO_2_, cells were overlaid with 1 × αMEM (Gibco) containing 0.2 mM L-glutamine (Gibco), 5% FBS (Gibco), 1 mM HEPES (Gibco), 1% Penicillin-Streptomycin (Gibco), and 1.25% carboxymethylcellulose sodium (Sigma), and incubated at 37°C with 5% CO_2_ for 48 hours. Plates were fixed with 4% PFA for at least 24 hours, rinsed with tap water, and stained with 1% crystal violet. The assay limit of detection (LOD) was 125 PFU/ g tissue for brain and foot and 50 PFU/mL for serum. Samples below the LOD were assigned a value half the LOD (brain and foot: 62.5 PFU/ g tissue; serum: 25 PFU/mL).

### *In vivo* bioluminescence imaging

Mice infected with CHIKV-nLuc as described above were imaged at 6 hours post-infection and on 1, 2, 3, 4, 5, and 7 DPI using the IVIS Spectrum multispectral *in vivo* imaging system (IVIS) as described previously [87]. Mice were anesthetized with 50 µL of ketamine (86 mg/kg) /xylazine (5 mg/kg) cocktail via intraperitoneal (IP) injection and injected retro-orbitally with 100 μL Nano-Glo substrate (Promega) diluted 1:10 in sterile PBS prior to imaging. Images were acquired using standardized exposure time, binning, and field-of-view settings. Both dorsal and ventral views were initially assessed, and consistent with previous findings by others [22], the ventral view produced equal-to-more intense signal (S9 Fig) and so was used for all subsequent experiments. IVIS was performed across four independent experiments. Images were analyzed using the manufacturer-recommended Aura Analysis Software (Spectral Instruments Imaging). Identical square regions of interest (ROIs) were applied to each mouse head using a standardized gating strategy that included the entire cranial area while carefully excluding the forepaws. nLuc intensity was quantified for both CC041 and B6 mice infected with either the nLuc or wild-type (WT) CHIKV LR strain, which served as the baseline control. For each experimental animal, raw nLuc values (two measurements per mouse per time point) were exported from Aura Analysis. Corresponding WT CHIKV LR controls, matched by mouse strain and sex where possible, were used to account for background nLuc. The nLuc intensity of each nLuc-infected mouse was corrected by subtracting the value of its matched WT CHIKV LR control. The two measurements per mouse were then averaged to obtain a single mean nLuc value per time point. To facilitate visualization of relative changes in signal intensity across mouse strains, background-corrected nLuc values were log_10_-transformed before analysis.

### IFN-α and IFN-β measurements

IFN‑α and IFN-β levels in left foot homogenates and serum were quantified using the ProcartaPlex Mouse IFN‑alpha/IFN‑beta Panel (Invitrogen, EPX02A-22187-901) according to the manufacturer’s instructions in a BSL‑3 facility with an established decontamination protocol. Following the addition of Streptavidin‑PE and subsequent plate washes, assay plates were decontaminated with freshly prepared 4% PFA for 30 min at room temperature (RT). The plates were then transferred to a BSL‑2 facility, washed three additional times with wash buffer, and the remaining steps of the manufacturer’s protocol were completed. Cytokines were analyzed on a Luminex 200 Analyzer instrument and quantified by comparison to a standard curve. All reported analyte values correspond to Luminex-derived concentrations calculated from individual calibration curves for each analyte. The assay LOD for IFN-α was 14.65 pg / g tissue for foot homogenates and 2.93 pg / mL for sera, and for IFN-β was 36.25 pg / g tissue for foot homogenates and 7.25 pg / mL for sera. Samples that fell below the assay LOD were assigned a value of half of the LOD (foot homogenates: 7.33 pg / g for IFN-α, 18.13 pg / g for IFN-β, sera: 1.47 pg / mL for IFN-α, 3.63 pg / mL for IFN-β).

### RNA isolation and quantitative polymerase chain reaction

One mL of TRIzol (Invitrogen) was added to homogenized foot pellets (after removing clarified homogenate for infectious virus quantification as stated above) and homogenized twice at 60Hz for one minute each using a MagNA Lyser (Roche). Samples were then centrifuged at 15,000 × g for 5 min at 4ºC, and then the clarified supernatant was transferred to another tube. RNA was isolated using the RNeasy Tissue Mini Kit (Qiagen) with a chloroform separation according to the manufacturer’s protocol as described previously [20]. A 15 min DNase I (Qiagen) digest was used to purify samples. cDNA was generated using 1 µg of RNA and the high-capacity cDNA reverse transcription kit (Applied Biosystems) according to the manufacturer’s protocol. For the qPCR reaction, the TaqMan Fast Advanced Master Mix (ThermoFisher) was used according to the manufacturer’s protocol. An amount of 4 μL of cDNA and 1 μL of primer probe (S4 Table) were added to each reaction. The reaction was run for 40 cycles using the QuantStudio 5 (Applied Biosystems). All samples that fell below the limit of detection were assigned a Ct value of 40 for all primer probe sets. All Ct values were normalized to *Gapdh*, and data were reported as fold change (2^−∆∆Ct^) compared to mock-infected samples from each mouse strain (CC041 and B6).

CHIKV viral RNA was quantified by qPCR in the cDNA prepared above using primers and a probe specific to the CHIKV E1 gene (S5 Table). Standard curves were made by making 10-fold serial dilutions of Synthetic CHIKV RNA (VR-3246SD, ATCC) and Rodent *Gapdh* (4308313, Applied Biosystems), and CHIKV E1 and *Gapdh* copies in each sample were calculated using the standard curves. CHIKV RNA copies were normalized to *Gapdh* copies and reported as Log(CHIKV E1 copies / 10^6^ *Gapdh* copies).

### Cytokine and chemokine quantification by Luminex

Cytokines and chemokines were quantified in ipsilateral left foot and serum samples using the BioPlex Pro Mouse Cytokines 23-Plex assay (Bio-Rad) according to the manufacturer’s protocol. Briefly, 50µL of clarified left foot samples or 1:4 diluted serum samples were used and incubated with magnetic capture beads, detection antibodies, and SA-PE with washes in between. The Bio-Plex System (Bio-Rad) was used to acquire cytokine levels by standard curve. IL-6, IL-9, and IL-13 were not detectable in samples, so they were excluded from analyses.

### Immunohistochemistry

Mouse tissue sections for IHC staining were cut to 4µm thickness, mounted onto positively charged slides, and allowed to air-dry overnight. For single chromogen CHIKV IHC, slides were loaded onto the Discovery Ultra IHC/ISH automatic stainer, and deparaffinization occurred using Discovery Wash (Roche cat. # 950-510). Cell conditioning occurred using Discovery CC1 (Roche cat. # 950-500) at 95°C for 64 min. The blocking of endogenous peroxidase was performed using Discovery Inhibitor (Roche cat. # 760-4840) for 8 min. Slides were then incubated with CHIKV rabbit polyclonal antibody (1:500 dilution, Novus Biologicals cat. # NBP3-13436) for 1 hour at RT. Detection was performed using Anti-rabbit HQ (Roche cat. # 760-4815) for 8 min followed by Anti-HQ HRP (Roche cat. # 760-4820) for 8 min at 36°C. CHIKV was then visualized using ChromoMAP DAB (Roche cat. # 760-159). The slides were counterstained using Hematoxylin (Roche cat. # 760-2021) for 8 min followed by Bluing Reagent (Roche cat. # 760-2037) for 4 min.

For the (3-plex) multiplex IHC involving OLIG2, CHIKV, and GFAP markers, slides were loaded onto the Roche Ventana Discovery Ultra IHC/ISH automated stainer. Deparaffinization occurred using Discovery Wash (Roche cat. # 950-510). Cell conditioning was performed using Discovery CC1 (Roche cat. # 950-500) at 95°C for 64 min. The blocking of endogenous peroxidase occurred using Discovery Inhibitor (Roche cat. # 760-4840) for 8 min. Slides were then incubated with OLIG2 (EP112) predilute rabbit monoclonal antibody (Roche cat. # 760-5050) for 16 min at 36°C. Detection was performed using Anti-rabbit HQ (Roche cat. # 760-4815) for 8 min at 36°C followed by anti-HQ HRP (Roche cat. # 760-4820) for 8 min at 36°C. OLIG2 (EP112) was visualized by applying Discovery Yellow HRP chromogen kit (Roche cat. # 760-250) for 64 min. Slides were then denatured using ULTRA Cell Conditioning Solution (ULTRA CC2, Roche cat. # 950-223) at 95°C for 8 min. Next, slides were incubated with CHIKV rabbit polyclonal antibody (1:500 dilution, Novus Biologicals cat. # NBP3-13436) for 1 hour at RT. Detection was performed using anti-rabbit HQ for 8 min at 36°C followed by anti-HQ HRP for 8 min at 36°C. Visualization of CHIKV was achieved by applying Discovery Teal HRP chromogen kit (Roche cat. # 760-247) for 32 min. Slides were denatured a second time using ULTRA CC2 at 95°C for 8 min and then incubated with anti-GFAP rabbit polyclonal antibody (1:1000 dilution, Sigma-Aldrich cat. # AB5804) for 1 hour at RT. Detection was performed using anti-rabbit HQ for 8 min at 36°C followed by anti-HQ HRP for 8 min at 36°C. Visualization was achieved by applying Discovery Purple chromogen kit (Roche cat. # 760-229) to the slides for 32 min. The slides were counterstained using Hematoxylin (Roche cat. # 760-2021) for 4 min followed by Bluing Reagent (Roche cat. # 760-2037) for 4 min. Infected oligodendrocytes and astrocytes were quantified using the HALO system, and the percentage of cells infected was calculated as the number of CHIKV+ cells divided by the total number of cells for each cell type, multiplied by 100.

For the (4-plex) multiplex IHC involving SOX-10, CHIKV, IBA1, and NeuN markers, slides were loaded onto the Roche Ventana Discovery Ultra IHC/ISH automated stainer. Deparaffinization occurred using Discovery Wash (Roche cat. # 950-510). Cell conditioning was performed using Discovery CC1 (Roche cat. # 950-500) at 95°C for 64 min. The blocking of endogenous peroxidase occurred using Discovery Inhibitor (Roche cat. # 760-4840) for 8 min. Slides were then incubated with SOX-10 (SP267) rabbit monoclonal antibody (Roche cat. # 760-4968) for 32 min at 36°C. Detection was performed using anti-rabbit HQ (Roche cat. # 760-4815) for 8 min at 36°C followed by anti-HQ HRP (Roche cat. # 760-4820) for 8 min at 36°C. SOX-10 (SP267) was visualized by applying Discovery Yellow HRP chromogen kit (Roche cat. # 760-250) for 72 min. Slides were then denatured using ULTRA Cell Conditioning Solution (ULTRA CC2, Roche cat. # 950-223) at 95°C for 8 min. Next, slides were incubated with CHIKV rabbit polyclonal antibody (1:500 dilution, Novus Biologicals cat. # NBP3-13436) for 1 hour at RT. Detection was performed using anti-rabbit HQ for 8 min at 36°C followed by anti-HQ HRP for 8 min at 36°C. Visualization of CHIKV was achieved by applying Discovery Teal HRP chromogen kit (Roche cat. # 760-247) for 32 min. Slides were denatured a second time using ULTRA CC2 at 95°C for 8 min and then incubated with IBA1 rabbit polyclonal antibody (1:800 dilution, Proteintech cat. # 10904-1-AP) for 1 hour at RT. Detection was performed using anti-rabbit HQ for 8 min at 36°C followed by anti-HQ HRP for 8 min at 36°C. IBA1 visualization was achieved by applying Discovery Purple chromogen kit (Roche cat. # 760-229) to the slides for 32 min. Slides were denatured a third time using ULTRA CC2 at 95°C for 8 min and then incubated with anti-NeuN rabbit polyclonal antibody (1:500 dilution, Abcam cat. # ab104225) for 1 hour at RT. Detection was performed using anti-rabbit HQ for 8 min at 36°C followed by anti-HQ HRP for 8 min at 36°C. Visualization of NeuN was achieved by applying Discovery ChromoMap DAB chromogen (Roche cat. # 760-159). The slides were counterstained using Hematoxylin (Roche cat. # 760-2021) for 4 min followed by Bluing Reagent (Roche cat. # 760-2037) for 4 min.

### Blood-brain barrier permeability assay

Following CHIKV infection as described above, at 2 and 5 DPI, mice received a 100 µL IP injection of 100mg/mL NaF diluted in sterile PBS. After 45 min, mice were euthanized and perfused as described above. Left brain hemispheres were homogenized to 20% w/v as described above and 100µL of undiluted homogenate was pipetted in duplicate into a black walled, clear-bottomed plate that contained NaF standards ranging 10-0.3125 µg/mL. All procedures were done with the biosafety cabinet light off. NaF was then quantified using the GloMax 3500 (excitation 475nm, emission 500–550nm). Protein concentration was measured using a bicinchoninic acid (BCA) assay per manufacturer protocol (Pierce). NaF levels were normalized to total protein and presented as µg of NaF in tissue/mg of protein.

### Monocyte/macrophage depletion

Macrophages were depleted using clodronate liposome (Liposoma; CP-010-010). Two days prior to infection, mice received 200 μL of clodronate liposomes or control liposomes via IP injection. Monocyte depletion was confirmed on the day of infection by peripheral blood smear and monocyte enumeration (S2 and S3 Tables). A 100 μL booster dose of liposome was administered on 2 DPI. Mice were euthanized at 5 DPI, and the brain, spleen, and left foot were collected. CHIKV brain titers were quantified by plaque assay using the left-brain hemispheres as described above.

### Biostatistical analyses

Statistical analyses were performed using GraphPad Prism 9. Biological replicates (individual mice) are indicated in figure legends. Viral titers and nLuc measurements were analyzed by two-way ANOVA with Sidak’s or Tukey’s multiple comparisons tests. All observations were independent and the assumptions of variance were met. Data were log_10_ transformed where necessary to meet assumptions of normality. A p-value of < 0.05 was considered statistically significant.

## Supporting information

S1 Fig Brain titers in Collaborative Cross (CC) mice following peripheral CHIKV infection. CHIKV brain titers were measured by plaque assay at 5 DPI in ten CC lines (A) and in CC007 (B) and CC041 (C) mice over time following SQ FP inoculation with 10^3^ PFU CHIKV SL165649. Plaque assay limit of detection (LOD) is depicted by the horizontal dotted line; all samples that fell below the plaque assay LOD were assigned a value of half of the LOD.
(TIF)

S2 Fig Effect of CHIKV input dose on brain infection in CC041 mice. Brain (A, D), foot (B, E), and serum (C, F) viral titers were measured by plaque assay at 5 DPI following SQ FP inoculation with 10^3^ PFU (square symbols) or 10^5^ PFU (triangular symbols) of CHIKV LR (A-C, red symbols) or CHIKV SL15649 (D-F, blue symbols) in CC041 mice. Assay limit of detection (LOD) is depicted by the horizontal dotted line; all samples that fell below the plaque assay LOD were assigned a value of half of the LOD. Note: The 10^3^ PFU titers presented in this figure are the same data as presented in Fig 1.
(TIF)

S3 Fig Viral load, IFN-β, and cytokine/chemokine gene expression CHIKV-infected CC041 and B6 mice. CC041 and B6 mice were infected with 10^3^ PFU of CHIKV LR (A, B, E) or SL15649 (C, D), and feet and sera were collected following euthanasia. (A,B) Infectious CHIKV LR in CC041 (red squares) and B6 (black circles) mouse feet (A) and sera (B) was quantified by plaque assay. (C,D) IFN-β in CHIKV SL15649-infected CC041 (bright blue squares) and B6 (navy circles) mouse feet (C) and sera (D) was quantified by Luminex. E) RNA was extracted from mock-infected or CHIKV LR-infected CC041 (red squares) and B6 (black circles) mouse feet, and *Ifna4, Ifnb1, Ccl2, Il10, Tnf, Ifng,* and *Il6* expression were quantified by qPCR. Assay limit of detection (LOD) is depicted by the horizontal dotted line; all samples that fell below the plaque assay LOD were assigned a value of half of the LOD; * p < 0.05; ** p < 0.01, Šidák’s multiple comparisons.
(TIF)

S4 Fig Proinflammatory cytokine and chemokine levels in CHIKV-infected CC041 and B6 feet and sera. CC041 (red squares) and B6 (black circles) mice were infected with 10^3^ PFU of CHIKV LR, and feet and sera were collected following euthanasia. Cytokine and chemokine levels were quantified in feet (A) and sera (B) by Luminex. Assay limit of detection (LOD) is depicted by the horizontal dotted line; all samples that fell below the plaque assay LOD were assigned a value of half of the LOD; * p < 0.05; ** p < 0.01, ***p < 0.001, ****p < 0.001, Šidák’s multiple comparisons.
(TIF)

S5 Fig Evaluating potential mechanisms of neuroinvasion in CHIKV-infected CC041 mice. A) Mice were mock-infected with PBS (open symbols) or infected with 10^3^ PFU of CHIKV LR (closed symbols) and intraperitoneally injected with sodium fluorescein (NaF) prior to euthanasia at 2 DPI, and NaF and total protein were quantified in 20% w/v brain homogenates. B,C) Clinical scores in CC041 mice injected with clodronate (B) or control (C) liposomes at -2 DPI and infected with 10^3^ PFU of CHIKV-nLuc via SQ FP. Clinical scores are represented as follows: 0 = clinically normal; 1 = mild abnormal gait and tail posture; 2 = hunched posture, abnormal gait, unkempt hair coat; 3 = marked hunched posture, and decreased ambulation (humane endpoint); 4 = moribund/dead/euthanized.
(TIF)

S6 Fig Clinical disease evaluation in CHIKV-infected B6 mice. B6 mice were infected with 10^3^ PFU CHIKV LR (n = 8) or mock-infected (n = 8) with PBS SQ via FP. A) Mock-infected (open symbols) and CHIKV-infected (closed symbols) mice were weighed daily. B,C) Clinical scores in mock-infected (B) and CHIKV-infected (C) B6 mice were assigned to each mouse daily as follows: 0 = clinically normal; 1 = mild abnormal gait and tail posture; 2 = hunched posture, abnormal gait, unkempt hair coat; 3 = marked hunched posture, and decreased ambulation (humane endpoint); 4 = moribund/dead/euthanized. During daily clinical observations, qualitative descriptors were recorded, including facial grimace, hyperactivity, abnormal behaviors (e.g., digging, excessive grooming), altered gait or posture (e.g., wide-based gait, wide rear-limb posture), and abnormal tail carriage (e.g., stiff-tail, tail-flagging). D) Spider plot illustrating relative frequency of the qualitative descriptors observed in CHIKV LR- (red shading) and mock-infected (black shading) B6 mice. Data are presented as the mean SEM; in (A), the horizontal dotted line represents the 0 DPI value to which values at all subsequent timepoints were normalized.
(TIF)

S7 Fig Representative image of abnormal tail carriage seen in CHIKV LR-infected CC041 mice. (TIF)

S8 Fig CHIKV titers in CC041 mouse brains at later timepoints. Brain viral titers were measured by plaque assay at 10, 14, and 28 DPI following SQ FP inoculation with 10^3^ CHIKV LR in CC041 mice. Assay limit of detection (LOD) is depicted by the horizontal dotted line; all samples that fell below the plaque assay LOD were assigned a value of half of the LOD.
(TIF)

S9 Fig Comparison of ventral and dorsal imaging approaches. Representative IVIS images of ventral (top images) and dorsal (bottom images) views of CC041 mice infected with CHIKV-nLuc- (left images) or CHIKV-WT- (right images) at 5 DPI.
(TIF)

S1 Video Representative clinical signs in CHIKV LR-infected CC041 mice. (MP4)

S2 Video Representative clinical signs in mock-infected CC041 mice. (MP4)

S1 Table Gene expression in mock-infected CC041 and B6 mouse feet. ΔCt values calculated by taking the difference between target gene Ct values and *Gapdh* Ct values for each mouse. Data presented as mean ± SD. P-values calculated by unpaired t-test.
(TIFF)

S2 Table Testing monocyte depletion following clodronate liposome dose. Blood was collected from B6 mice immediately before the first dose of liposomes was administered to establish baseline numbers. Blood smears were performed at baseline, 24, 48 and 96 hours. Mice were re-dosed at 48 hours with 100µL (mouse 1) or 200µL (mouse 2) with clodronate liposomes. Total monocytes were counted out of 100 cells across multiple fields. Counts were performed in triplicate.
(TIFF)

S3 Table Confirmation of monocyte depletion following clodronate liposome administration. Blood smears were performed from blood collected from CC041 mice immediately before CHIKV infection and at 48 hours after clodronate or control liposome administration to confirm depletion. Total monocytes were counted out of 100 cells across multiple fields. Counts were performed in triplicate.
(TIFF)

S4 Table Primers and Probes. (TIFF)

S5 Table CHIKV RNA Primers and Probes. (TIFF)

## References

[1] Puntasecca CJ, King CH, LaBeaud AD. Measuring the global burden of chikungunya and Zika viruses: a systematic review. PLoS Negl Trop Dis. 2021;15(3):e0009055. doi: 10.1371/journal.pntd.0009055 33661908 PMC7932082

[2] European Centre for Disease Prevention and Control. Chikungunya virus disease worldwide overview. European Centre for Disease Prevention and Control. Available from: https://www.ecdc.europa.eu/en/chikungunya-monthly. Accessed 2025 August 5.

[3] Center for Disease Prevention. Areas at risk for chikungunya. Available from: https://www.cdc.gov/chikungunya/data-maps/index.html. Accessed 2025 August 5.

[4] Goldstein J. New York confirms state’s first locally acquired case of chikungunya. N Y Times. 2025.

[5] Lantz AM, Baxter VK. Neuropathogenesis of Old world alphaviruses: considerations for the development of medical countermeasures. Viruses. 2025;17(2):261. doi: 10.3390/v17020261 40007016 PMC11860675

[6] Mehta R, Gerardin P, de Brito CAA, Soares CN, Ferreira MLB, Solomon T. The neurological complications of chikungunya virus: a systematic review. Rev Med Virol. 2018;28(3):e1978. doi: 10.1002/rmv.1978 29671914 PMC5969245

[7] Cerny T, Schwarz M, Schwarz U, Lemant J, Gérardin P, Keller E. The range of neurological complications in chikungunya fever. Neurocrit Care. 2017;27(3):447–57. doi: 10.1007/s12028-017-0413-8 28741102

[8] Chen LH, Fritzer A, Hochreiter R, Dubischar K, Meyer S. From bench to clinic: the development of VLA1553/IXCHIQ, a live-attenuated chikungunya vaccine. J Travel Med. 2024;31(7):taae123. doi: 10.1093/jtm/taae123 39255380 PMC11497415

[9] FDA update on the safety of Ixchiq (Chikungunya vaccine, live). FDA Suspends Biologics License; Medical Product Safety Information; 2025.

[10] Richardson JS, Anderson DM, Mendy J, Tindale LC, Muhammad S, Loreth T, et al. Chikungunya virus virus-like particle vaccine safety and immunogenicity in adolescents and adults in the USA: a phase 3, randomised, double-blind, placebo-controlled trial. Lancet Lond Engl. 2025;405:1343–52. doi: 10.1016/S0140-6736(25)00345-9 40158526

[11] Tindale LC, Richardson JS, Anderson DM, Mendy J, Muhammad S, Loreth T, et al. Chikungunya virus virus-like particle vaccine safety and immunogenicity in adults older than 65 years: a phase 3, randomised, double-blind, placebo-controlled trial. Lancet Lond Engl. 2025;405:1353–61. doi: 10.1016/S0140-6736(25)00372-1 40158524

[12] Hucke FIL, Bugert JJ. Current and promising antivirals against chikungunya virus. Front Public Health. 2020;8:618624. doi: 10.3389/fpubh.2020.618624 33384981 PMC7769948

[13] Palha N, Guivel-Benhassine F, Briolat V, Lutfalla G, Sourisseau M, Ellett F, et al. Real-time whole-body visualization of Chikungunya Virus infection and host interferon response in zebrafish. PLoS Pathog. 2013;9(9):e1003619. doi: 10.1371/journal.ppat.1003619 24039582 PMC3764224

[14] Passoni G, Langevin C, Palha N, Mounce BC, Briolat V, Affaticati P, et al. Imaging of viral neuroinvasion in the zebrafish reveals that Sindbis and chikungunya viruses favour different entry routes. Dis Model Mech. 2017;10(7):847–57. doi: 10.1242/dmm.029231 28483796 PMC5536907

[15] Inglis FM, Lee KM, Chiu KB, Purcell OM, Didier PJ, Russell-Lodrigue K, et al. Neuropathogenesis of Chikungunya infection: astrogliosis and innate immune activation. J Neurovirol. 2016;22(2):140–8. doi: 10.1007/s13365-015-0378-3 26419894 PMC4783292

[16] Labadie K, Larcher T, Joubert C, Mannioui A, Delache B, Brochard P, et al. Chikungunya disease in nonhuman primates involves long-term viral persistence in macrophages. J Clin Invest. 2010;120(3):894–906. doi: 10.1172/JCI40104 20179353 PMC2827953

[17] Workman AD, Charvet CJ, Clancy B, Darlington RB, Finlay BL. Modeling transformations of neurodevelopmental sequences across mammalian species. J Neurosci. 2013;33(17):7368–83. doi: 10.1523/JNEUROSCI.5746-12.2013 23616543 PMC3928428

[18] Churchill GA, Airey DC, Allayee H, Angel JM, Attie AD, Beatty J, et al. The collaborative cross, a community resource for the genetic analysis of complex traits. Nat Genet. 2004;36(11):1133–7. doi: 10.1038/ng1104-1133 15514660

[19] Collaborative Cross Consortium. The genome architecture of the collaborative cross mouse genetic reference population. Genetics. 2012;190(2):389–401. doi: 10.1534/genetics.111.132639 22345608 PMC3276630

[20] Anderson EJ, Knight AC, Heise MT, Baxter VK. Effect of Viral strain and host age on clinical disease and viral replication in immunocompetent mouse models of chikungunya encephalomyelitis. Viruses. 2023;15(5):1057. doi: 10.3390/v15051057 37243143 PMC10220978

[21] Woodson CM, Carney SK, Kehn-Hall K. Neuropathogenesis of encephalitic alphaviruses in non-human primate and mouse models of infection. Pathogens. 2025;14(2):193. doi: 10.3390/pathogens14020193 40005568 PMC11858634

[22] Sun C, Gardner CL, Watson AM, Ryman KD, Klimstra WB. Stable, high-level expression of reporter proteins from improved alphavirus expression vectors to track replication and dissemination during encephalitic and arthritogenic disease. J Virol. 2014;88(4):2035–46. doi: 10.1128/JVI.02990-13 24307590 PMC3911548

[23] Carpentier KS, Morrison TE. Innate immune control of alphavirus infection. Curr Opin Virol. 2018;28:53–60. doi: 10.1016/j.coviro.2017.11.006 29175515 PMC5835171

[24] Cook LE, Locke MC, Young AR, Monte K, Hedberg ML, Shimak RM, et al. Distinct roles of interferon alpha and beta in controlling chikungunya virus replication and modulating neutrophil-mediated inflammation. J Virol. 2019;94(1):e00841-19. doi: 10.1128/JVI.00841-19 31619554 PMC6912113

[25] de Souza WM, Fumagalli MJ, de Lima STS, Parise PL, Carvalho DCM, Hernandez C, et al. Pathophysiology of chikungunya virus infection associated with fatal outcomes. Cell Host Microbe. 2024;32(4):606-622.e8. doi: 10.1016/j.chom.2024.02.011 38479396 PMC11018361

[26] Das T, Hoarau JJ, Bandjee MCJ, Maquart M, Gasque P. Multifaceted innate immune responses engaged by astrocytes, microglia and resident dendritic cells against Chikungunya neuroinfection. J Gen Virol. 2015;96(Pt 2):294–310. doi: 10.1099/vir.0.071175-0 25351727

[27] Wei Chiam C, Fun Chan Y, Chai Ong K, Thong Wong K, Sam I-C. Neurovirulence comparison of chikungunya virus isolates of the Asian and East/Central/South African genotypes from Malaysia. J Gen Virol. 2015;96:3243–54. doi: 10.1099/jgv.0.000263 26276497

[28] McRae M. HIV and viral protein effects on the blood brain barrier. Tissue Barriers. 2016;4(1):e1143543. doi: 10.1080/21688370.2016.1143543 27141423 PMC4836474

[29] Li F, Wang Y, Yu L, Cao S, Wang K, Yuan J, et al. Viral Infection of the central nervous system and neuroinflammation precede blood-brain barrier disruption during japanese encephalitis virus infection. J Virol. 2015;89(10):5602–14. doi: 10.1128/JVI.00143-15 25762733 PMC4442524

[30] Mustafá YM, Meuren LM, Coelho SVA, de Arruda LB. Pathways exploited by flaviviruses to counteract the blood-brain barrier and invade the central nervous system. Front Microbiol. 2019;10:525. doi: 10.3389/fmicb.2019.00525 30984122 PMC6447710

[31] Morrison TE, Oko L, Montgomery SA, Whitmore AC, Lotstein AR, Gunn BM, et al. A mouse model of chikungunya virus–induced musculoskeletal inflammatory disease. Am J Pathol. 2011;178:32–40. doi: 10.1016/j.ajpath.2010.11.018 21224040 PMC3069999

[32] Gardner J, Anraku I, Le TT, Larcher T, Major L, Roques P, et al. Chikungunya virus arthritis in adult wild-type mice. J Virol. 2010;84(16):8021–32. doi: 10.1128/JVI.02603-09 20519386 PMC2916516

[33] Couderc T, Chrétien F, Schilte C, Disson O, Brigitte M, Guivel-Benhassine F, et al. A mouse model for Chikungunya: young age and inefficient type-I interferon signaling are risk factors for severe disease. PLoS Pathog. 2008;4(2):e29. doi: 10.1371/journal.ppat.0040029 18282093 PMC2242832

[34] Priya R, Patro IK, Parida MM. TLR3 mediated innate immune response in mice brain following infection with Chikungunya virus. Virus Res. 2014;189:194–205. doi: 10.1016/j.virusres.2014.05.010 24905288

[35] Constant LEC, Rajsfus BF, Carneiro PH, Sisnande T, Mohana-Borges R, Allonso D. Overview on chikungunya virus infection: from epidemiology to state-of-the-art experimental models. Front Microbiol. 2021;12:744164. doi: 10.3389/fmicb.2021.744164 34675908 PMC8524093

[36] Qadri SW, Kumar N, Santhoshkumar R, Desai A, Ravi V, Venkataswamy MM. Infection of human microglial cell line CHME-3 to study neuropathogenesis of chikungunya virus. J Neurovirol. 2022;28(3):374–82. doi: 10.1007/s13365-022-01070-7 35352315

[37] de Lima Cavalcanti TYV, Pereira MR, de Paula SO, Franca RF de O. A review on chikungunya virus epidemiology, pathogenesis and current vaccine development. Viruses. 2022;14(5):969. doi: 10.3390/v14050969 35632709 PMC9147731

[38] Ferris MT, Aylor DL, Bottomly D, Whitmore AC, Aicher LD, Bell TA, et al. Modeling host genetic regulation of influenza pathogenesis in the collaborative cross. PLoS Pathog. 2013;9(2):e1003196. doi: 10.1371/journal.ppat.1003196 23468633 PMC3585141

[39] Gralinski LE, Ferris MT, Aylor DL, Whitmore AC, Green R, Frieman MB, et al. Genome wide identification of SARS-CoV susceptibility loci using the collaborative cross. PLoS Genet. 2015;11(10):e1005504. doi: 10.1371/journal.pgen.1005504 26452100 PMC4599853

[40] Chaaithanya IK, Muruganandam N, Anwesh M, Rajesh R, Ghosal SR, Kartick C, et al. HLA class II allele polymorphism in an outbreak of chikungunya fever in Middle Andaman, India. Immunology. 2013;140(2):202–10. doi: 10.1111/imm.12128 23710940 PMC3784166

[41] Cvejic E, Lemon J, Hickie IB, Lloyd AR, Vollmer-Conna U. Neurocognitive disturbances associated with acute infectious mononucleosis, Ross River fever and Q fever: a preliminary investigation of inflammatory and genetic correlates. Brain Behav Immun. 2014;36:207–14. doi: 10.1016/j.bbi.2013.11.002 24211375

[42] Chaaithanya IK, Muruganandam N, Surya P, Anwesh M, Alagarasu K, Vijayachari P. Association of oligoadenylate synthetase gene cluster and DC-SIGN (CD209) gene polymorphisms with clinical symptoms in chikungunya virus infection. DNA Cell Biol. 2016;35(1):44–50. doi: 10.1089/dna.2015.2819 26398832

[43] Dutta SK, Tripathi A. Association of toll-like receptor polymorphisms with susceptibility to chikungunya virus infection. Virology. 2017;511:207–13. doi: 10.1016/j.virol.2017.08.009 28888110

[44] Sengupta S, Mukherjee S, Bhattacharya N, Tripathi A. Differential genotypic signatures of toll-like receptor polymorphisms among dengue-chikungunya mono- and co-infected eastern Indian patients. Eur J Clin Microbiol Infect Dis. 2021;40:1369–81. doi: 10.1007/s10096-020-04125-x 33495940

[45] Sengupta S, Bhattacharya N, Tripathi A. Association of C-reactive protein polymorphisms with serum-CRP concentration and viral load among dengue-chikungunya mono/co-infected patients. Antiviral Res. 2022;197:105225. doi: 10.1016/j.antiviral.2021.105225 34915091

[46] Schoenrock SA, Gaines CH, Kumar P, Khan S, Farrington J, Ferris MT, et al. Genetic mapping in collaborative cross mouse strains identifies loci that affect initial sensitivity to cocaine. Psychopharmacology (Berl). 2026;243(6):1471–88. doi: 10.1007/s00213-025-06901-z 41143948 PMC12617008

[47] Yang H, Wang X, Wang P, He L, Schick SF, Jacob P 3rd, et al. Thirdhand tobacco smoke exposure increases the genetic background-dependent risk of pan-tumor development in Collaborative Cross mice. Environ Int. 2023;174:107876. doi: 10.1016/j.envint.2023.107876 36940581 PMC11439420

[48] Tran TDB, Monroy Hernandez C, Nguyen H, Wright S, Center for Systems Neurogenetics of Addiction, Tarantino LM, et al. The microbial community dynamics of cocaine sensitization in two behaviorally divergent strains of collaborative cross mice. Genes Brain Behav. 2023;22(3):e12845. doi: 10.1111/gbb.12845 37114320 PMC10242200

[49] Hopkins HK, Traverse EM, Barr KL. Chikungunya encephalitis: an inconsistently reported headache and cause of death in patients with pre-existing conditions. Current Tropical Medicine Reports. 2022;9:73–91. doi: 10.1007/s40475-022-00258-5

[50] Jain J, Narayanan V, Kumar A, Shrinet J, Srivastava P, Chaturvedi S, et al. Establishment and comparison of pathogenicity and related neurotropism in two age groups of immune competent mice, C57BL/6J using an indian isolate of chikungunya virus (CHIKV). Viruses. 2019;11(6):578. doi: 10.3390/v11060578 31242674 PMC6631960

[51] Graham VA, Easterbrook L, Rayner E, Findlay-Wilson S, Flett L, Kennedy E, et al. Comparison of chikungunya virus-induced disease progression and pathogenesis in type-i interferon receptor-deficient mice (A129) and two wild-type (129Sv/Ev and C57BL/6) mouse strains. Viruses. 2024;16(10):1534. doi: 10.3390/v16101534 39459867 PMC11512278

[52] Reynaud JM, Kim DY, Atasheva S, Rasalouskaya A, White JP, Diamond MS, et al. IFIT1 Differentially interferes with translation and replication of alphavirus genomes and promotes induction of type I interferon. PLoS Pathog. 2015;11(4):e1004863. doi: 10.1371/journal.ppat.1004863 25927359 PMC4415776

[53] Poddar S, Hyde JL, Gorman MJ, Farzan M, Diamond MS. The interferon-stimulated gene IFITM3 restricts infection and pathogenesis of arthritogenic and encephalitic alphaviruses. J Virol. 2016;90(19):8780–94. doi: 10.1128/JVI.00655-16 27440901 PMC5021394

[54] Schilte C, Couderc T, Chretien F, Sourisseau M, Gangneux N, Guivel-Benhassine F, et al. Type I IFN controls chikungunya virus via its action on nonhematopoietic cells. J Exp Med. 2010;207(2):429–42. doi: 10.1084/jem.20090851 20123960 PMC2822618

[55] Kalra P, Kister B, Fendt R, Köster M, Pulverer J, Sahle S, et al. A comparative computational analysis of IFN-alpha pharmacokinetics and its induced cellular response in mice and humans. PLoS Comput Biol. 2025;21(9):e1013509. doi: 10.1371/journal.pcbi.1013509 40997098 PMC12500084

[56] Ryman KD, Klimstra WB, Nguyen KB, Biron CA, Johnston RE. Alpha/beta interferon protects adult mice from fatal Sindbis virus infection and is an important determinant of cell and tissue tropism. J Virol. 2000;74(7):3366–78. doi: 10.1128/jvi.74.7.3366-3378.2000 10708454 PMC111838

[57] Grieder FB, Vogel SN. Role of interferon and interferon regulatory factors in early protection against Venezuelan equine encephalitis virus infection. Virology. 1999;257(1):106–18. doi: 10.1006/viro.1999.9662 10208925

[58] Baxter VK, Griffin DE. Interferon gamma modulation of disease manifestation and the local antibody response to alphavirus encephalomyelitis. J Gen Virol. 2016;97(11):2908–25. doi: 10.1099/jgv.0.000613 27667782 PMC5770845

[59] Hampton BK, Plante KS, Whitmore AC, Linnertz CL, Madden EA, Noll KE, et al. Forward genetic screen of homeostatic antibody levels in the Collaborative Cross identifies MBD1 as a novel regulator of B cell homeostasis. PLoS Genet. 2022;18(12):e1010548. doi: 10.1371/journal.pgen.1010548 36574452 PMC9829176

[60] Graham JB, Swarts JL, Leist SR, Schäfer A, Bell TA, Hock P, et al. Unique immune profiles in collaborative cross mice linked to survival and viral clearance upon infection. iScience. 2024;27(3):109103. doi: 10.1016/j.isci.2024.109103 38361611 PMC10867580

[61] Casiraghi C, Dorovini-Zis K, Horwitz MS. Epstein-Barr virus infection of human brain microvessel endothelial cells: a novel role in multiple sclerosis. J Neuroimmunol. 2011;230(1–2):173–7. doi: 10.1016/j.jneuroim.2010.08.003 20826008

[62] Fletcher NF, Wilson GK, Murray J, Hu K, Lewis A, Reynolds GM, et al. Hepatitis C virus infects the endothelial cells of the blood-brain barrier. Gastroenterology. 2012;142(3):634-643.e6. doi: 10.1053/j.gastro.2011.11.028 22138189 PMC3801216

[63] Xu Z, Waeckerlin R, Urbanowski MD, van Marle G, Hobman TC. West Nile virus infection causes endocytosis of a specific subset of tight junction membrane proteins. PLoS One. 2012;7(5):e37886. doi: 10.1371/journal.pone.0037886 22655077 PMC3359987

[64] Ohka S, Nihei C, Yamazaki M, Nomoto A. Poliovirus trafficking toward central nervous system via human poliovirus receptor-dependent and -independent pathway. Fron Microbiol. 2012;3:147. doi: 10.3389/fmicb.2012.00147 PMC3328850 22529845

[65] Dups J, Middleton D, Yamada M, Monaghan P, Long F, Robinson R, et al. A new model for Hendra virus encephalitis in the mouse. PLoS One. 2012;7(7):e40308. doi: 10.1371/journal.pone.0040308 22808132 PMC3393746

[66] Tirabassi RS, Townley RA, Eldridge MG, Enquist LW. Molecular mechanisms of neurotropic herpesvirus invasion and spread in the CNS. Neurosci Biobehav Rev. 1998;22(6):709–20. doi: 10.1016/s0149-7634(98)00009-8 9809306

[67] Kaul M, Garden GA, Lipton SA. Pathways to neuronal injury and apoptosis in HIV-associated dementia. Nature. 2001;410(6831):988–94. doi: 10.1038/35073667 11309629

[68] Boothpur R, Brennan DC. Human polyoma viruses and disease with emphasis on clinical BK and JC. J Clin Virol. 2010;47(4):306–12. doi: 10.1016/j.jcv.2009.12.006 20060360 PMC3774018

[69] Salimi H, Cain MD, Jiang X, Roth RA, Beatty WL, Sun C, et al. Encephalitic alphaviruses exploit caveola-mediated transcytosis at the blood-brain barrier for central nervous system entry. mBio. 2020;11(1):e02731-19. doi: 10.1128/mBio.02731-19 32047126 PMC7018649

[70] Alvarez PA, Tang A, Winters DM, Kaushal P, Medina A, Nieto MV, et al. Old World alphaviruses use distinct mechanisms to infect brain microvascular endothelial cells for neuroinvasion. Cell Rep. 2025;44(10):116305. doi: 10.1016/j.celrep.2025.116305 40987287 PMC12617940

[71] Robin S, Ramful D, Le Seach’ F, Jaffar-Bandjee M-C, Rigou G, Alessandri J-L. Neurologic manifestations of pediatric chikungunya infection. J Child Neurol. 2008;23(9):1028–35. doi: 10.1177/0883073808314151 18287573

[72] Potter MC, Baxter VK, Mathey RW, Alt J, Rojas C, Griffin DE, et al. Neurological sequelae induced by alphavirus infection of the CNS are attenuated by treatment with the glutamine antagonist 6-diazo-5-oxo-l-norleucine. J Neurovirol. 2015;21:159–73. doi: 10.1007/s13365-015-0314-6 25645378 PMC4375032

[73] Earnest MP, Goolishian HA, Calverley JR, Hayes RO, Hill HR. Neurologic, intellectual, and psychologic sequelae following Western encephalitis. Neurology. 1971;21(9):969. doi: 10.1212/WNL.21.9.969 5106260

[74] Crawley JN. What’s wrong with my mouse? Behavioral phenotyping of transgenic and knockout mice. 2nd ed. Hoboken, NJ, US: John Wiley & Sons, Inc.; 2007.

[75] Metcalf TU, Griffin DE. Alphavirus-induced encephalomyelitis: antibody-secreting cells and viral clearance from the nervous system. J Virol. 2011;85(0):11490–501. doi: 10.1128/JVI.05379-11 21865385 PMC3194963

[76] Miller KD, Matullo CM, Milora KA, Williams RM, O’Regan KJ, Rall GF. Immune-mediated control of a dormant neurotropic RNA virus infection. J Virol. 2019;93(18):e00241-19. doi: 10.1128/JVI.00241-19 31270232 PMC6714802

[77] Baxter VK, Griffin DE. Interferon-gamma modulation of the local T cell response to alphavirus encephalomyelitis. Viruses. 2020;12(1):113. doi: 10.3390/v12010113 31963302 PMC7019780

[78] Fragkoudis R, Dixon-Ballany CM, Zagrajek AK, Kedzierski L, Fazakerley JK. Following acute encephalitis, semliki forest virus is undetectable in the brain by infectivity assays but functional virus RNA capable of generating infectious virus persists for life. Viruses. 2018;10(5):273. doi: 10.3390/v10050273 29783708 PMC5977266

[79] Ireland DDC, Manangeeswaran M, Lewkowicz AP, Engel K, Clark SM, Laniyan A, et al. Long-term persistence of infectious Zika virus: inflammation and behavioral sequela in mice. PLoS Pathog. 2020;16(12):e1008689. doi: 10.1371/journal.ppat.1008689 33301527 PMC7728251

[80] Figueiredo CP, Barros-Aragão FGQ, Neris RLS, Frost PS, Soares C, Souza INO, et al. Zika virus replicates in adult human brain tissue and impairs synapses and memory in mice. Nat Commun. 2019;10(1):3890. doi: 10.1038/s41467-019-11866-7 31488835 PMC6728367

[81] Knight AC, Nagel J, Anderson EJ, Atkins HM, Montgomery S, Baxter VK. Neurological chikungunya virus infection induces corpus callosum degeneration associated with resident immune cell activation and peripheral immune cell infiltration. J Immunol. 2022;208. doi: 10.4049/jimmunol.208.Supp.126.22

[82] Griffin DE, Johnson RT. Role of the immune response in recovery from Sindbis virus encephalitis in mice. J Immunol. 1977;118(3):1070–5. 845432

[83] Jackson AC, Moench TR, Trapp BD, Griffin DE. Basis of neurovirulence in Sindbis virus encephalomyelitis of mice. Lab Invest. 1988;58(5):503–9. 3367635

[84] Baxter VK, Glowinski R, Braxton AM, Potter MC, Slusher BS, Griffin DE. Glutamine antagonist-mediated immune suppression decreases pathology but delays virus clearance in mice during nonfatal alphavirus encephalomyelitis. Virology. 2017;508:134–49. doi: 10.1016/j.virol.2017.05.013 28531865 PMC5510753

[85] Jackson AC, Moench TR, Griffin DE, Johnson RT. The pathogenesis of spinal cord involvement in the encephalomyelitis of mice caused by neuroadapted Sindbis virus infection. Lab Invest. 1987;56(4):418–23. 3031369

[86] Canto MCD, Rabinowitz SG. Central nervous system demyelination in Venezuelan equine encephalomyelitis infection: an experimental model of virus-induced myelin injury. J Neurol Sci. 1981;49:397–418. doi: 10.1016/0022-510X(81)90030-7 7217991

[87] Barre RS, Mostafa A, Chiem K, Pearl RL, Platt RN, Cupic A, et al. Bioluminescent reporter influenza A viruses to track viral infections. Microbiol Spectr. 2025;13(11):e0215025. doi: 10.1128/spectrum.02150-25 41060012 PMC12584733

