# Supplementary figures and images for: Characterization of an immunocompetent, young adult mouse model for studying chikungunya virus neuroinvasion and central nervous system infection
Source: PLoS Pathog. 2026 Jul 10;22(7):e1014395. doi: 10.1371/journal.ppat.1014395 (PMC13379094; doi:10.1371/journal.ppat.1014395)

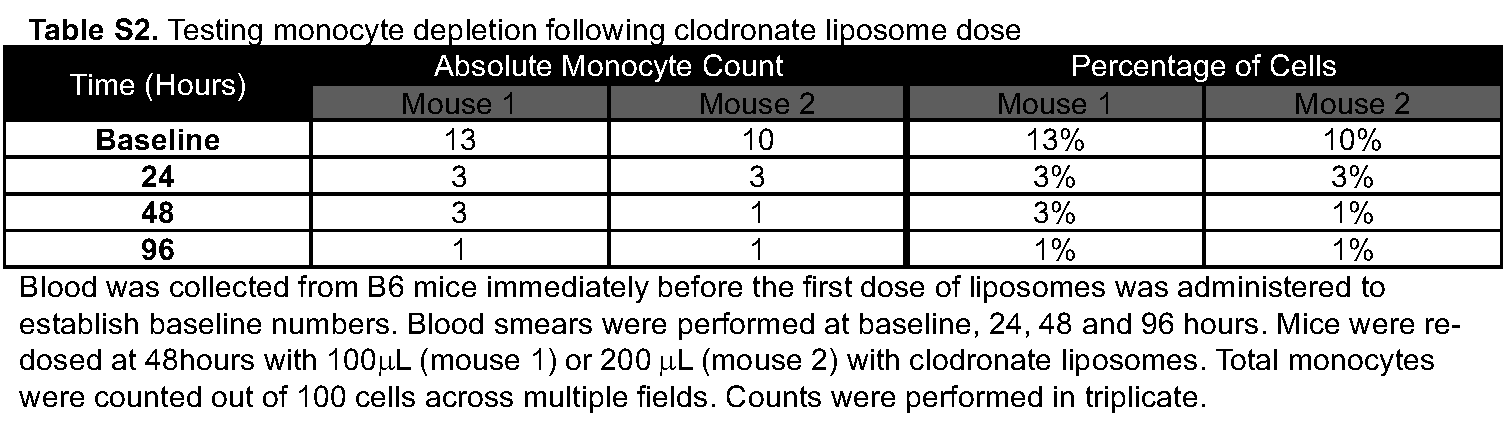

Supplement: S2 Table — Blood was collected from B6 mice immediately before the first dose of liposomes was administered to establish baseline numbers. Blood smears were performed at baseline, 24, 48 and 96 hours. Mice were re-dosed at 48 hours with 100µL (mouse 1) or 200µL (mouse 2) with clodronate liposomes. Total monocytes were counted out of 100 cells across multiple fields. Counts were performed in triplicate. (TIFF) [file ppat.1014395.s013.tiff]

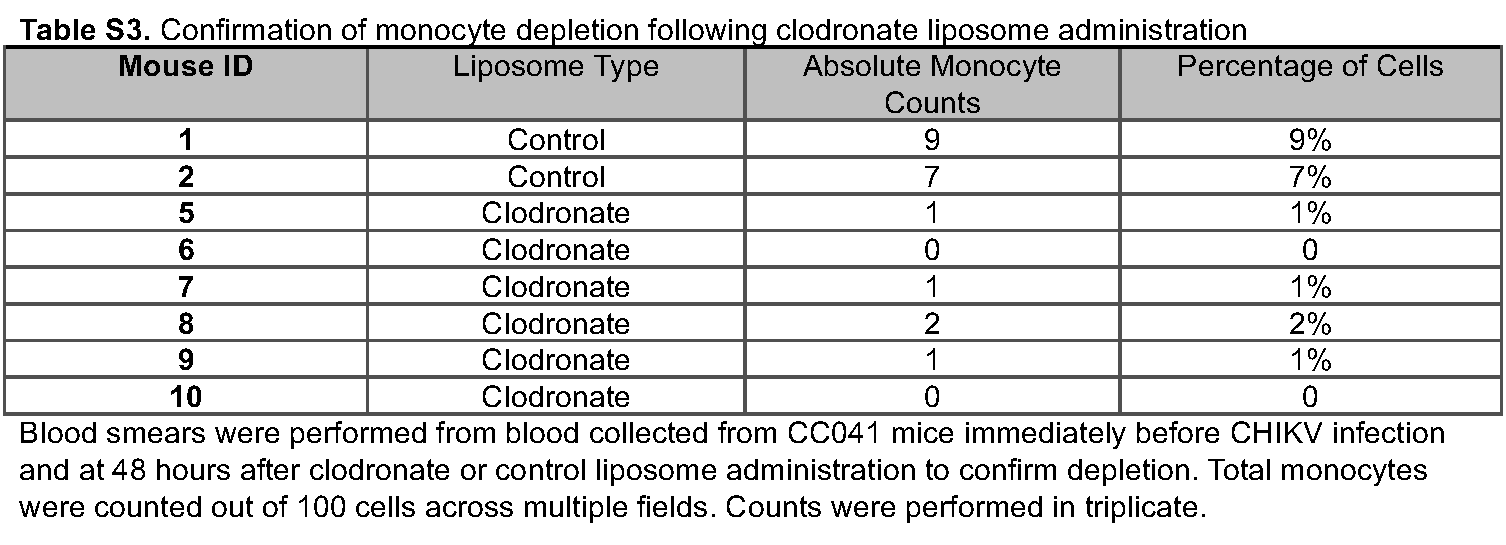

Supplement: S3 Table — Blood smears were performed from blood collected from CC041 mice immediately before CHIKV infection and at 48 hours after clodronate or control liposome administration to confirm depletion. Total monocytes were counted out of 100 cells across multiple fields. Counts were performed in triplicate. (TIFF) [file ppat.1014395.s014.tiff]

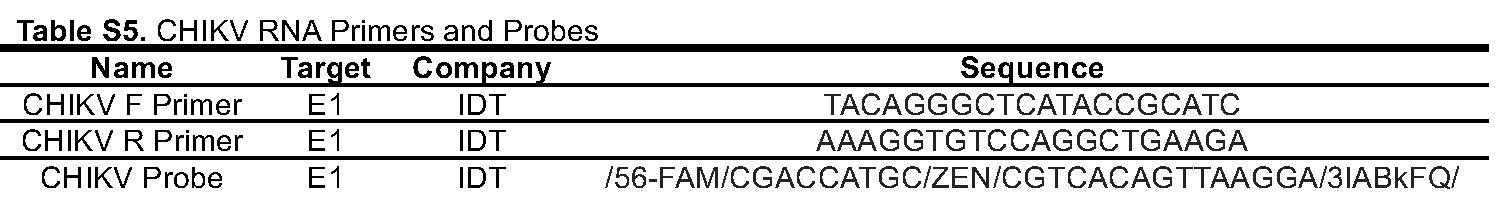

Supplement: S5 Table — (TIFF) [file ppat.1014395.s016.tiff]
